# A Case Study of the Glycoside Hydrolase Enzyme Mechanism Using an Automated QM-Cluster Model Building Toolkit

**DOI:** 10.3389/fchem.2022.854318

**Published:** 2022-03-24

**Authors:** Qianyi Cheng, Nathan John DeYonker

**Affiliations:** Department of Chemistry, University of Memphis, Memphis, TN, United States

**Keywords:** glycoside hydrolase (GH), QM-cluster models, glycosylation, density functional (DFT), residue interaction network, computational enzymology

## Abstract

Glycoside hydrolase enzymes are important for hydrolyzing the β-1,4 glycosidic bond in polysaccharides for deconstruction of carbohydrates. The two-step retaining reaction mechanism of Glycoside Hydrolase Family 7 (GH7) was explored with different sized QM-cluster models built by the Residue Interaction Network ResidUe Selector (RINRUS) software using both the wild-type protein and its E217Q mutant. The first step is the glycosylation, in which the acidic residue 217 donates a proton to the glycosidic oxygen leading to bond cleavage. In the subsequent deglycosylation step, one water molecule migrates into the active site and attacks the anomeric carbon. Residue interaction-based QM-cluster models lead to reliable structural and energetic results for proposed glycoside hydrolase mechanisms. The free energies of activation for glycosylation in the largest QM-cluster models were predicted to be 19.5 and 31.4 kcal mol^−1^ for the wild-type protein and its E217Q mutant, which agree with experimental trends that mutation of the acidic residue Glu217 to Gln will slow down the reaction; and are higher in free energy than the deglycosylation transition states (13.8 and 25.5 kcal mol^−1^ for the wild-type protein and its mutant, respectively). For the mutated protein, glycosylation led to a low-energy product. This thermodynamic sink may correspond to the intermediate state which was isolated in the X-ray crystal structure. Hence, the glycosylation is validated to be the rate-limiting step in both the wild-type and mutated enzyme.

## Introduction

The organic biopolymer cellulose, which acts as a large source of carbohydrates, is found abundantly in the cell wall of green plants (Updegraff, 1969; Klemm et al., 2005). Cellulose consists of hundreds to thousands of β-1,4 linked glucose units, which can be cleaved to form shorter chains by degradation processes. Glycoside hydrolase (GH) enzymes, as one of many enzyme classes that can modify, decompose, and assemble carbohydrates in nature (Cantarel et al., 2009), are responsible for breaking down the glycosidic β-1,4 linkages between C_1_-O_4_ *via* a hydrolysis reaction. Two mechanisms have been proposed for the reaction, “retaining” and “inverting” (Koshland, 1953). Utilized by many GH families (Cantarel et al., 2009), the retaining mechanism consists of two steps (Sulzenbacher et al., 1997; Davies et al., 1998; Mackenzie et al., 1998). In step one, a proton will be transferred from an acidic residue to the glycosidic oxygen (O_4_), coupling with a nucleophilic attack of the anomeric carbon of the carbohydrate (C_1_) forming a glycosyl-enzyme intermediate (GEI). In step two, a water molecule attacks the anomeric carbon, breaking the GEI bond and transferring a proton back to the acidic residue. These two steps are also respectively called glycosylation and deglycosylation. In the less-common inverting mechanism, proton transfer causes the cleavage of the glycosidic bond to take place *via* nucleophilic attack by water (Koshland, 1953). The focus of this study will be exploring the retaining mechanism, which is utilized by many GHs, specifically GH Family 7 (GH7). Note that a protein in the GH7 family can be interchangeably called Cellobiohydrolase 7A (Cel7A), Exocellobiohydrolase I (CBHI), and 1,4-β-cellobiohydrolase, though the recommended name is Exoglucanase 1 to adhere to the Enzyme Commission number EC:3.2.1.91.

GH7 cellobiohydrolases depolymerize single chains from crystalline cellulose and hydrolyze cellobiose units from the reducing end (Divne et al., 1994; Chundawat et al., 2011). GH7 cellobiohydrolases are major components of enzyme cocktails for biodegrading plant material (Martinez et al., 2008) and biofuels processes in industry (Chundawat et al., 2011). Therefore, numerous studies have been carried out focusing on the structures, reaction mechanisms, and energetics of GH7 cellobiohydrolases (Divne et al., 1994; Divne et al., 1998; Bu et al., 2011; Chundawat et al., 2011; Kern et al., 2013; Knott et al., 2014a; Knott et al., 2014b; Vermaas et al., 2015; Knott et al., 2020). The first structure of a cellulase was determined in 1994 from the *Trichoderma reesei* extracellular cellobiohydrolase I (CBHI, *Tr*Cel7A, an anamorph of the fungus *Hypocrea jecorina*) (Divne et al., 1994). *Tr*Cel7A binds 10 glucosyl units (+3 to −7, positive and negative numbers indicating the reducing and nonreducing end of the cellulose chain) (Davies et al., 1997). It was found to catalyze the hydrolysis of the glycosidic β-1,4 linkage specifically (breaking the bond between the +1 and −1 units) in cellulose *via* a proposed retaining mechanism (Wolfenden et al., 1998). This bond is difficult to hydrolyze without a catalyst, for example the free energy of activation has been measured to be 36.8 ± 2.2 kcal/mol at pH 10.6 and 25°C for *β*-methylglucopyranoside in potassium acetate, phosphate or carbonate buffer (Wolfenden et al., 1998).

The glycosyl substrate distortion in the hydrolysis of glycosides has been studied extensively (Vocadlo and Davies, 2008; Mayes et al., 2014). The IUPAC conventions for labelling ring conformations are used throughout (Conformational Nomenclature for Five and Six-Membered Ring Forms of Monosaccharides and Their Derivatives. Recommendations 1980, IUPAC-IUB, 1980). Large conformational change of the −1 glycose unit from the half-chair, envelope, or boat-like conformation to the ^*4*^ *C*_*1*_ chair conformation is typically observed in the glycosylation step (Vocadlo and Davies, 2008). This was also observed in a recent study by Beckham, Ståhlberg, Withers, Götz, *et al.* (Knott et al., 2014b), that the −1 glucose unit adopts a ^*4*^ *E* envelope conformation in the Michaelis complex of the *H. jecorina* Cel7A (*Hje*Cel7A) E217Q mutated protein and an intact cellononaose chain (PDB: 4C4C) and a ^*4*^ *C*_*1*_ chair conformation in the GEI; which was further confirmed in molecular dynamics (MD) simulations using the theoretical model of the wild-type enzyme (PDB: 8CEL, a Michaelis complex of the protein and 10 glucosyl units). The MD simulations suggested that the glucose unit changed from a half-chair (^*4*^ *H*_*5*_) in the Michaelis complex to a half-chair (^*4*^ *H*_*3*_) at the transition state, then to a stable chair (^*4*^ *C*_*1*_) conformation in the glycosylation product state (Knott et al., 2014b). The transition state geometries were also found to be able to adopt the ^*4*^ *H*_*3*_ and ^*3*^ *H*_*4*_ half-chair and ^*2,5*^ *B* and *B*_*2,5*_ boat conformations (Cremer and Pople, 1975; Davies et al., 2003; Vocadlo and Davies, 2008; Satoh and Manabe, 2013).

In an early mutation study of *Tr*Cel7A, the catalytic activities (*k*_*cat*_) were measured to be 12.8 ± 0.2, 0.15 ± 0.05, 0.035 ± 0.005, and 0.0063 ± 0.0005 min^−1^ at pH 5.7 and 37°C for the wild-type, the D214E, E212Q and E217Q mutated proteins, respectively. These values correspond to a free energy of activation of 19.1 kcal mol^−1^ for the wild-type protein and 23.8 kcal mol^−1^ for the E217Q mutated protein (Ståhlberg et al., 1996), indicating the E217Q mutation slowed down the reaction. An experimental study using high-speed atomic force microscopy found that the rate of processive cellobiose hydrolysis by *Hje*Cel7A on a crystalline cellulose surface was 7.1 ± 3.9 s^−1^ at 30°C (16.3–17.1 kcal mol^−1^) (Igarashi et al., 2011). In a more recent study comparing another GH7 from the fungus *Penicillium funiculosum* with *Tr*Cel7A, the *k*_*cat*_ for the E217Q mutated protein was reported to be 14.26 ± 1.05 min^−1^ at pH = 4 and 55°C (20.2–20.3 kcal mol^−1^) (Taylor et al., 2018). This rate constant is also slower than that of the wild-type protein.

In the study by Beckham, Ståhlberg, Withers, Götz, *et al.* (Knott et al., 2014b), the mixed quantum mechanics/molecular mechanics (QM/MM) and transition path sampling method was applied to study the full retaining catalytic mechanism starting from the Michaelis complex of theoretical model PDB: 8CEL and 10 glucosyl units (+2 to −7, Glc452 to Glc460) at 300 K and 1.0 bar (Knott et al., 2014b). The simulation results indicated that in the glycosylation step, the nucleophile Glu212, which first hydrogen bonds with Asp214, attacked the anomeric carbon C_1_ on the −1 position (Glc454) of glucopyranose, breaking the C_1_-O_4_ bond. A proton on the acidic residue Glu217 transferred to O_4_ on the +1 position (Glc453) of glucopyranose. In the deglycosylation step, one water enters the active site, undergoes nucleophilic attack on the −1 glycosyl unit bonding to the C_1_, breaks the bond between the glucopyranose and Glu212 oxygen, and lastly donates its proton to Glu217. The QM/MM computations predicted that the glycosylation was the rate limiting step with a free energy of activation of 15.5 kcal mol^−1^, and the intermediate structure was 2.5 kcal mol^−1^ lower in energy than the initial reactant. For deglycosylation, the free energy of activation of the elementary step was 11.6 kcal mol^−1^, and the final product was 2.1 kcal mol^−1^ lower in free energy than the initial reactant.

The glycosylation of GH7 (PDB: 8CEL) was later studied by the Wang group using both a pure quantum mechanics (QM) and QM/MM approach (Li et al., 2010). The activation free energy was computed to be 14.1 kcal mol^−1^ using a density functional theory (DFT)-based QM-cluster model at the B3LYP/6-31G(d, p) level of theory. The QM-cluster model was very small, only including residues E212, E217, +1 and −1Glc. A higher free energy glycosylation TS (23.9 kcal mol^−1^) was found when using QM/MM in the same study (Li et al., 2010). The relatively high QM/MM glycosylation barrier was explained as arising from the catalytic residues frozen in the MM-region, and this indicated that the active site environment has an important impact on the kinetics (Li et al., 2010). In the same study, deglycosylation was examined by adding one water molecule to the QM-cluster model and a much higher free energy of activation, 32.9 kcal mol^−1^ at B3LYP/6-31G(d,p) level of theory, was computed for the glycosylation (Li et al., 2010). The results of Wang and coauthors suggest that the QM-region must be appropriately defined to obtain good agreement between experiment and theory for the GH7 enzyme.

Most early computational studies ignored deglycosylation, instead focusing on the rate-limiting glycosylation step. One full catalytic cycle was explored using a small model QM-cluster model (two residues and the substrate) built from the *Escherichia coli β*-galactosidase-galactopyranoside complex system with various DFT functionals and basis sets and found that the glycosylation activation energies ranged from 21.1 to 28.7 kcal mol^−1^ (Brás et al., 2008). Mutational effects were also studied with QM/MD simulation which predicted the glycosylation barriers to be 32.6 kcal mol^−1^ for wild-type *Tr*Cel7A (Yan et al., 2011). The following nucleophilic attack step had barriers of 0.4 kcal mol^−1^ for the elementary step (Yan et al., 2011). The glycosylation free energy barrier was computed to be 17.5 kcal mol^−1^ in a more recent DFTB/MM study on CBHI (Barnett et al., 2011), and 13.4 kcal mol^−1^ in a QM/MM metadynamics study on GH27 (Pan et al., 2013). Clearly, kinetics of various enzymes in the GHs family show variability, and the relationship between computational accuracy and partitioning of enzyme QM-regions needs to be explored further.

The workflow of the RINRUS (*Residue Interaction Network-based ResidUe Selector*) toolkit provides a standard QM-cluster model construction procedure, with embedded reproducibility for system comparison and benchmarking. The RINRUS software has been developed by our lab to rationally select and rank importance of biological fragments (amino acid residues, cofactors, solvent) for inclusion in QM-cluster models and write input files to set up QM calculations. Ranking of residue importance can be obtained through distance metrics, but also more importantly *via* qualitative topological features of the residue interaction network (Shannon et al., 2003; del Sol et al., 2006; Csermely, 2008; Vishveshwara et al., 2009; Doncheva et al., 2011; Di Paola et al., 2013) or from first-principles interaction energies computed *via* Symmetry Adapted Perturbation Theory (Summers et al., 2019). RINRUS has been applied to many other enzyme case studies which provided valuable results (DeYonker and Webster, 2013; DeYonker and Webster, 2015; Summers et al., 2018; Cheng and DeYonker, 2020; Summers et al., 2021; Cheng and DeYonker, 2021). It can currently interface with several quantum chemistry packages (such as Gaussian, Q-Chem, PSI4, and xtb). RINRUS is open source, but not yet publicly available. It is available by request from the authors and will be shared with the community once the web-based interface is refined and a peer-reviewed introduction to the software (currently in preparation) is published. Until public release of RINRUS is announced in the literature, a thorough description of its capabilities has been described in our work on calibrating QM-cluster models of catechol-*O*-methyltransferase (Summers et al., 2021).

In this study, we use RINRUS built QM-cluster models to study the full catalytic cycle of the retaining mechanism of cellulose hydrolysis based on the wild-type protein and its E217Q mutant. The proposed mechanistic detail and energetics will provide more insights for enzyme engineering of effective catalytic modifications.

## Computational Methods

Both a theoretical model of an exoglucanase 1 in complex with a cellulose nanomer (PDB ID: 8CEL) and the X-ray crystal structure of *Hypocrea jecorina* exoglucanase 1 E217Q in complex with a cellononaose (PDB ID: 4C4C, GH7) were used to construct the models for QM-cluster computations. Hydrogen atoms were added to backbone and side chain heavy atoms of the enzyme using the *reduce* program (Word et al., 1999b). Hydrogen atoms were also manually added to the cellononaose. The active site histidine residue (His228) was doubly protonated (X-ray crystal data for 4C4C is recorded at pH = 6). Next, the protonated PDB files were processed by *probe* (Word et al., 1999a) to generate the Residue Interaction Networks (RINs) based on atom contact information. In the RIN of 8CEL, 23 residues (including the Glc452, Glc453, Glc454, Glc455 as the +2, +1, −1, and −2 glucosyl units, 16 residues, and three waters) were identified by RINRUS that interact with the +1 and −1 glucosyl units (named Glc+1 and Glc−1, used as the RINRUS “seed” of our models), and 17 residues among the 23 have a high interaction score (more than 50 contact dot counts). Next, 22 residues (including the Bgc1, Bgc2, Bgc3 and Bgc4 as the +2, +1, −1, −2 glucosyl units, 14 residues, and four waters) were identified in the RIN of 4C4C, among which 17 fragments have a >50 interaction count with the seed. The catalytic triad residues Glu212, Asp214 and Glu217/GLN217 in 8CEL and 4C4C which were included in the QM region in the study by Beckham, Ståhlberg, Withers, Götz, *et al.* (Knott et al., 2014b), were all identified by RINRUS as they have high contact dot counts with the −1 or +1 glucosyl units. Glu212 is the nucleophile and is positioned closely to the −1 glucose with a C_1_(Glc454)-O(Glu212) bond distance of 3.44 Å in 8CEL and 3.40 Å in 4C4C. Glu217 and Asp214 were protonated to be neutral residues similar to the procedure in reference 13, Glu217 is the acidic residue, mutated to a protonated Gln in PDB:4C4C, that donates a proton to the +1 glucosyl unit, while Asp214 hydrogen bonds to Glu212 to support the catalytic reaction. All three residues exhibit multiple conformations in the X-ray crystal structure. In Conformation A of the PDB file, Gln217 is oriented towards the glycosidic bond (similar to Glu217 in 8CEL), therefore all the QM-cluster models were built based on this conformation.

**FIGURE 1:**
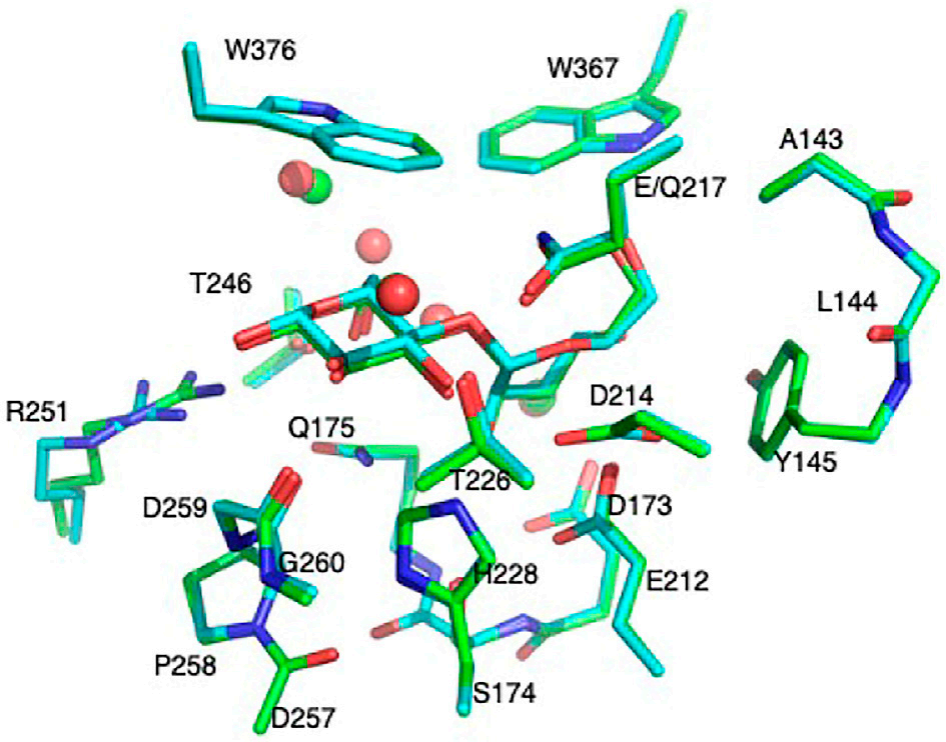
Aligned 21-residue model of 8CEL **Res21-E** (green, waters in green sphere) and 23- residue model of 4C4C **Res23-Q** (cyan, waters in red sphere).

**FIGURE 2:**
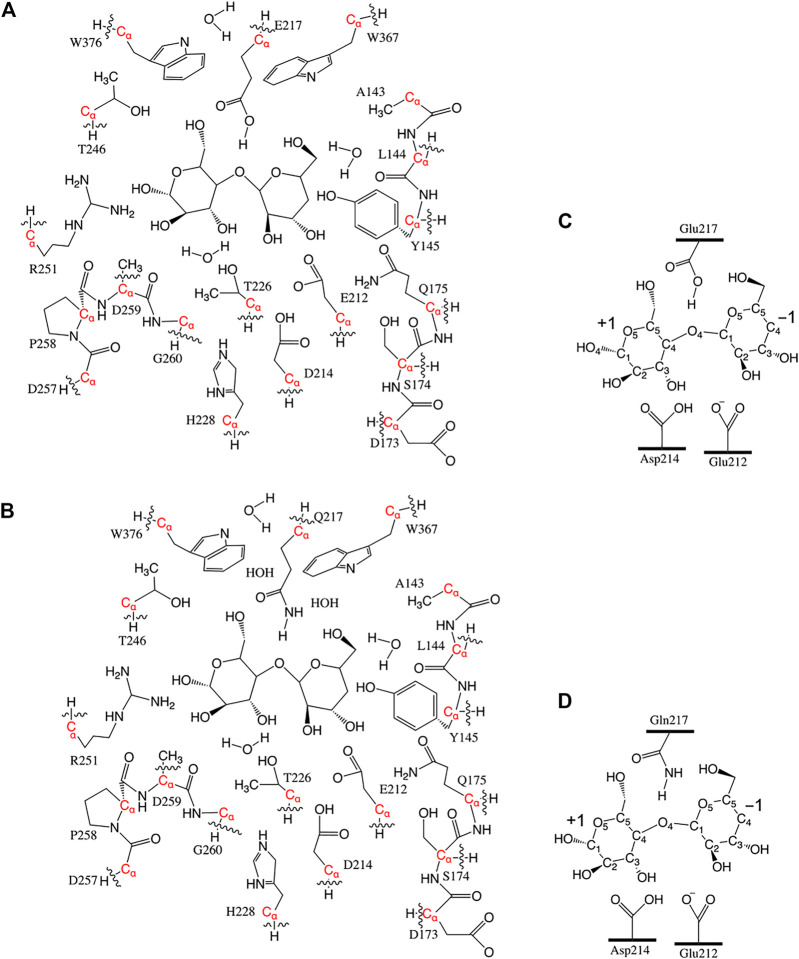
**(A)** 2D structure of the **Res21-E** (8CEL) model. The wavy lines indicate truncation of the residue at *C*_*α*_. **(B)** 2D structure of the **Res23-Q** (4C4C) model. The wavy lines indicate truncation of the residue at *C*_*α*_. Atom labeling scheme of +1 and −1 glucosyl units **(C)** in 8CEL and **(D)** in 4C4C.

Considering the glycosylation cleavage of the O_4_-C_1_ bond connecting the −1 and +1 glucosyl units, only the O_4_ atom in the glucosyl −2 unit was kept and protonated as a hydroxyl group in the model. The +2 unit was completely removed, and one hydrogen atom was added to the O_4_ atom of the −1 glucosyl unit. Hence, QM-cluster models of 16 residues (227 atoms, 12 *C*_*α*_ and 10 *C*_*β*_ atoms were kept frozen, named **Res16-E**) and 21 residues (the “maximal model” where all residues with nonzero contact dot counts with seed are included, 305 atoms, 19 *C*_*α*_ and 14 *C*_*β*_ atoms were kept frozen, named **Res21-E**) based on the 8CEL RIN were constructed. Frozen atoms in the QM-cluster models are provided in Table 1. A model from the same 16 residue set using PDB:4C4C was built with 228 atoms (same set of frozen atoms, named **Res16-Q**) and a “maximal model” that included all 20 residues identified by the RIN as having interactions with the seed (275 atoms, 14 *C*_*α*_ and nine *C*_*β*_ atoms were kept frozen, named **Res20-Q**). Residues Thr246 and Arg251 were included in the RIN of 8CEL but not 4C4C. In order to have some comparable analysis, two larger models were built by adding these residues to the 4C4C-based models (named **Res21-Q** which includes Arg251, and **Res23-Q** which includes Thr246, Arg251, and one additional water). The aligned **Res21-E** and **Res23-Q** 3D models are shown in Figure 1. The 2D structure of the **Res21-E** and **Res23-Q** model along with frozen atom information are shown in Figures 2A,B. The atomic labeling for the glycosyl units is shown in Figures 2C,D.

**TABLE 1:** Residue and trimming information for the various QM-cluster models built in this study.

	Residue name		Res16-E	Res21-E	Res16-Q	Res20-Q	Res21-Q	Res23-Q
	# of Atoms		227	305	228	275	297	312
		Charge	0	0	0	−1	0	0
1	Ala143	0	—	*C*_*α*_ *C*_*β*_	—	*C*_*α*_ *C*_*β*_	*C*_*α*_ *C*_*β*_	*C*_*α*_ *C*_*β*_
	*Leu144*	0	—	*C*_*α*_	—	*C*_*α*_	*C*_*α*_	*C*_*α*_
2	Tyr145	0	*C*_*α*_ *C*_*β*_	*C*_*α*_ *C*_*β*_	*C*_*α*_ *C*_*β*_	*C*_*α*_ *C*_*β*_	*C*_*α*_ *C*_*β*_	*C*_*α*_ *C*_*β*_
3	Asp173	−1	*C*_*α*_ *C*_*β*_	*C*_*α*_ *C*_*β*_	*C*_*α*_ *C*_*β*_	*C*_*α*_ *C*_*β*_	*C*_*α*_ *C*_*β*_	*C*_*α*_ *C*_*β*_
4	Ser174	0	—	*C*_*α*_ *C*_*β*_	—	*C*_*α*_ *C*_*β*_	*C*_*α*_ *C*_*β*_	*C*_*α*_ *C*_*β*_
5	Gln175	0	*C*_*α*_ *C*_*β*_	*C*_*α*_ *C*_*β*_	*C*_*α*_ *C*_*β*_	*C*_*α*_ *C*_*β*_	*C*_*α*_ *C*_*β*_	*C*_*α*_ *C*_*β*_
6	Glu212	−1	*C*_*α*_ *C*_*β*_	*C*_*α*_ *C*_*β*_	*C*_*α*_ *C*_*β*_	*C*_*α*_ *C*_*β*_	*C*_*α*_ *C*_*β*_	*C*_*α*_ *C*_*β*_
7	Asp214	0	*C*_*α*_ *C*_*β*_	*C*_*α*_ *C*_*β*_	*C*_*α*_ *C*_*β*_	*C*_*α*_ *C*_*β*_	*C*_*α*_ *C*_*β*_	*C*_*α*_ *C*_*β*_
8	Glu/Gln217	0/+1	*C*_*α*_ *C*_*β*_	*C*_*α*_ *C*_*β*_	*C*_*α*_ *C*_*β*_	*C*_*α*_ *C*_*β*_	*C*_*α*_ *C*_*β*_	*C*_*α*_ *C*_*β*_
9	Thr226	0	—	*C*_*α*_ *C*_*β*_	—	*C*_*α*_ *C*_*β*_	*C*_*α*_ *C*_*β*_	*C*_*α*_ *C*_*β*_
10	His228	+1	*C*_*α*_ *C*_*β*_	*C*_*α*_ *C*_*β*_	*C*_*α*_ *C*_*β*_	*C*_*α*_ *C*_*β*_	*C*_*α*_ *C*_*β*_	*C*_*α*_ *C*_*β*_
11	Thr246	0	—	*C*_*α*_ *C*_*β*_	—	—	—	*C*_*α*_ *C*_*β*_
12	Arg251	+1	*C*_*α*_ *C*_*β*_	*C*_*α*_ *C*_*β*_	*C*_*α*_ *C*_*β*_	—	*C*_*α*_ *C*_*β*_	*C*_*α*_ *C*_*β*_
	*Asp257*	−1	—	*C*_*α*_	—	*C*_*α*_	*C*_*α*_	*C*_*α*_
13	Pro258	0	—	*C*_*α*_	—	*C*_*α*_	*C*_*α*_	*C*_*α*_
14	Asp259	0	*C*_*α*_	*C*_*α*_	*C*_*α*_	*C*_*α*_	*C*_*α*_	*C*_*α*_
	*Gly260*	0	*C*_*α*_	*C*_*α*_	*C*_*α*_	*C*_*α*_	*C*_*α*_	*C*_*α*_
15	Trp367	0	*C*_*α*_ *C*_*β*_	*C*_*α*_ *C*_*β*_	*C*_*α*_ *C*_*β*_	*C*_*α*_ *C*_*β*_	*C*_*α*_ *C*_*β*_	*C*_*α*_ *C*_*β*_
16	Trp376	0	*C*_*α*_ *C*_*β*_	*C*_*α*_ *C*_*β*_	*C*_*α*_ *C*_*β*_	*C*_*α*_ *C*_*β*_	*C*_*α*_ *C*_*β*_	*C*_*α*_ *C*_*β*_
17	Glc+1	0	X	X	X	X	X	X
18	Glc–1	0	X	X	X	X	X	X
19	HOH1	0	X	X	X	X	X	X
20	HOH2	0	X	X	X	X	X	X
21	HOH3	0	—	X	—	X	X	X
22	HOH4	0	—	—	—	X	X	X
23	HOH5	0	—	—	—	—	—	X

″Cα″ indicates frozen alpha-carbon atoms for that residue, ″Cα Cβ″ indicates frozen alpha- and beta-carbons for that residue, ″X″ indicates the specified non-amino acid residue fragment is included in the model. Residues ID#s written in italics were not identified in the RIN as interacting with the seed, but were added as bridging residues connecting two discrete residues.

All quantum mechanical cluster model computations were performed using the Gaussian16 program (Frisch et al., 2016). Density functional theory (DFT) with the hybrid B3LYP exchange-correlation functional (Lee et al., 1988; Becke, 1993) was employed with the 6-31G(d′) basis set for N, O, and S atoms (Hariharan and Pople, 1973; Petersson and Al-Laham, 1991; Foresman and Frisch, 1996), and the 6-31G basis sets for C and H atoms (Hehre et al., 1972). QM-cluster models incorporated the Grimme D3 (Becke-Johnson) dispersion correction (GD3BJ) (Grimme et al., 2010; Grimme et al., 2011) and implicit solvation *via* the conductor-like polarizable continuum model (CPCM) (Barone and Cossi, 1998; Cossi et al., 2003) using universal force field (UFF) atomic radii, a non-default electrostatic scaling factor of 1.2, and the default parameters for water with an attenuated dielectric constant of ε = 4. This dielectric constant value has been previously determined as appropriate for simulating the less-polarized environment within an enzyme active site (Siegbahn and Blomberg, 2000; Blomberg et al., 2014). Unscaled harmonic vibrational frequency calculations were used to identify all stationary points as either minima (no imaginary frequencies) or transition states (TSs, only one imaginary frequency). TSs were located first for each elementary step of the proposed mechanism; the reactants and products were then located by following the intrinsic reaction coordinate (IRC) (Fukui, 1970; Fukui, 1981). It is important to note that our group uses the “freeze code” scheme in Gaussian16, where all Hessian elements are zero when involving frozen internal coordinates. The phenomenon where several small magnitude imaginary vibrational frequencies appear in the thermochemical analysis does not arise in our treatment of the Hessian matrix. Zero-point energies (ZPE) and thermal enthalpy/free energy corrections were computed at 1 atm and 310 K.

## Results and Discussion

The labeling scheme **ResV-W(X)-YZ** is used for the QM-cluster model illustrating the reaction mechanism, where **V** = # of residues in the model (16, 20, 21 or 23); **W** = E for residue E217 in the wild-type protein (8CEL) or Q for residue Q217 in the mutated protein (4C4C); **X** indicates when multiple conformations (**A, B or C**) are explored and reported; **Y** = R (reactant), TS (transition state), Int (intermediate state), or P (product); **Z** = 1 indicates glycosylation, **Z** = 2 indicates deglycosylation. For example, **Res16-EA-R1** refers to the optimized 16-residue glycosylation reactant with residue E217 (8CEL) in conformation A, which is the lowest energy reactant. The optimized structures (xyz-coordinates) of all the reactants, transition states, and products are in the SI (xyzfiles.zip).

Step 1 Glycosylation

**FIGURE 3:**
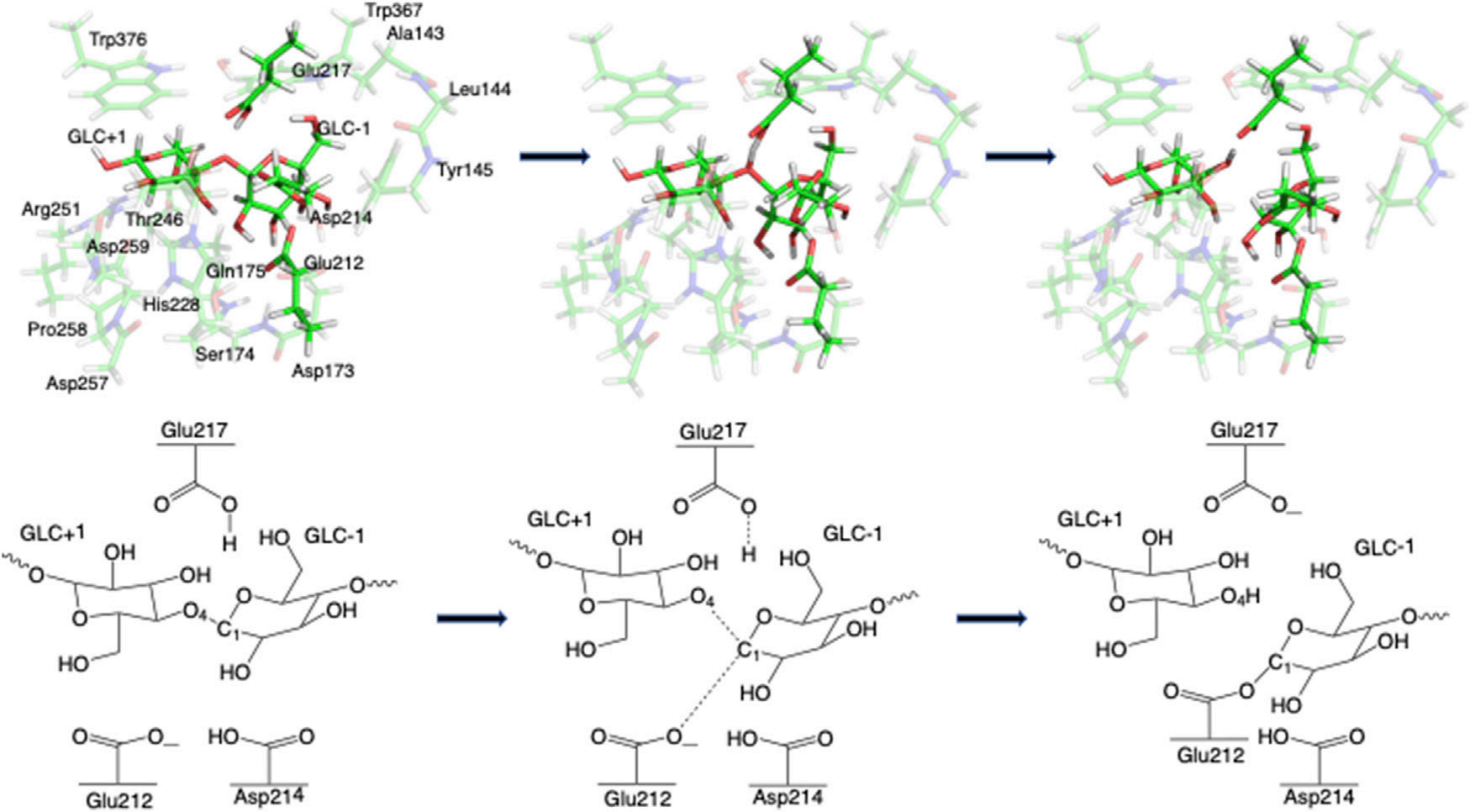
3D and 2D representation of glycosylation reaction mechanism of the **Res21-E** model of 8CEL.

**FIGURE 4:**
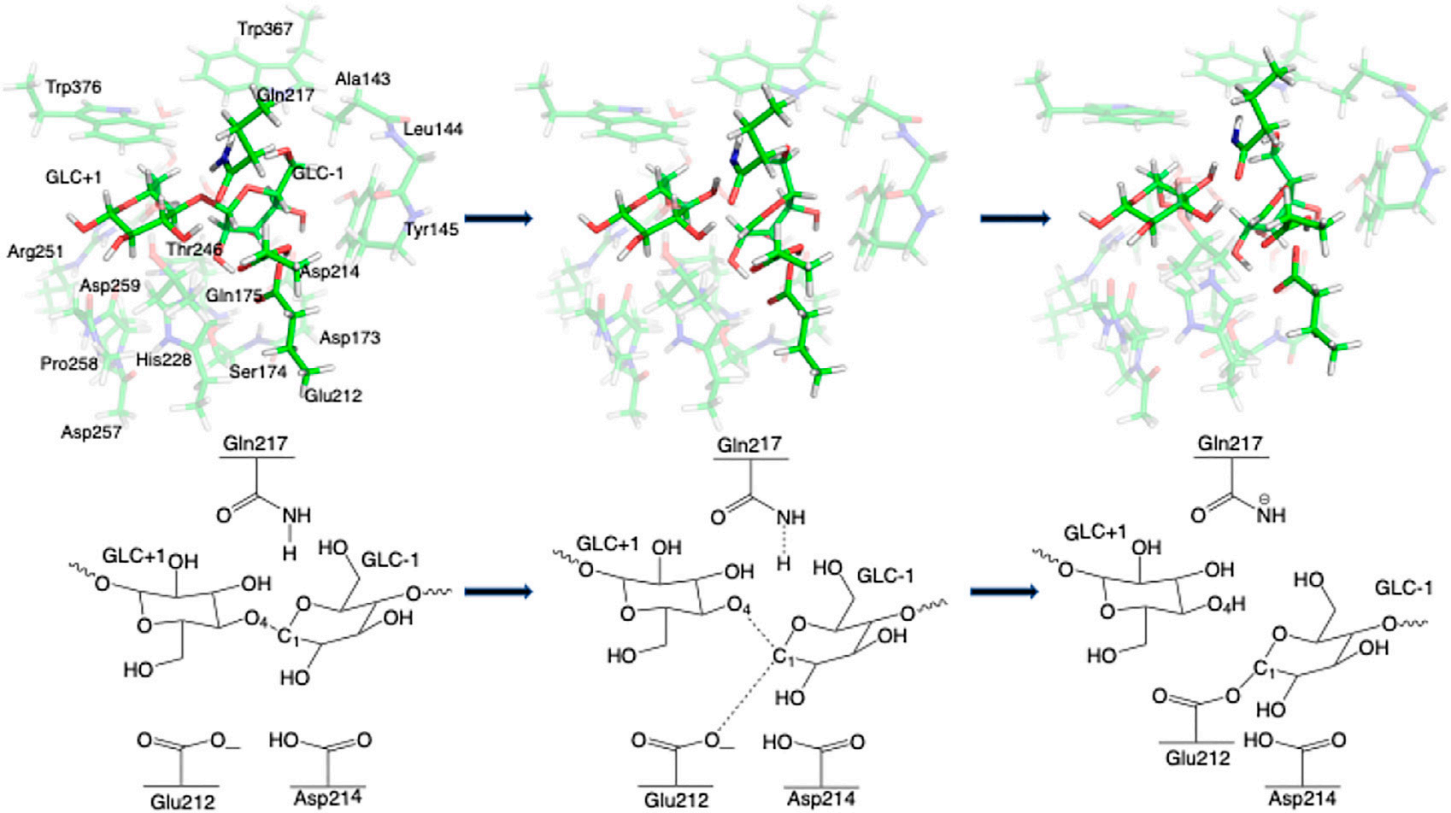
3D and 2D representation of glycosylation reaction mechanism of the **Res23-Q** model of 4C4C.

**FIGURE 5:**
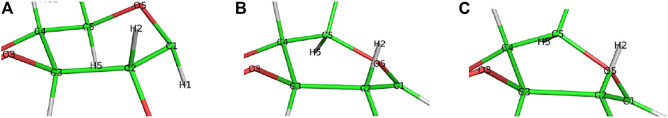
Conformational change of the glycosyl unit Glc−1 in the glycosylation reaction from **(A)** ^4^E in R1 to **(B)** ^4^H_3_ in TS1 to **(C)** ^4^C_1_ in P1.

The representative reactants, transition states (TS), and products of the glycosylation reaction step are shown in Figures 3, 4, which use the maximal model **Res21-E** (3D and 2D representation in Figure 3) and **Res23-Q** (3D and 2D representation in Figure 4) generated from the wild-type protein theoretical model (8CEL) and its E212Q mutant X-ray crystal structure (4C4C), respectively. The reactant is a Michaelis complex and the glycosidic bond (O_4_-C_1_) between Glc+1 and Glc−1 is intact. Glu217/Gln217 is protonated, and the proton is hydrogen bonding with O_4_ of Glc+1. In the TS, the proton of Glu217/Gln217 is transferred between the O_4_ of Glc+1 and sidechain oxygen atom of Glu217 or nitrogen atom of Glu217. While the glycosidic bond is elongated and the Glc−1 ring distorts from a ^*4*^ *E* envelope conformation in the reactant (R1) to a ^*4*^ *H*_*3*_ half chair conformation (in TS1), the anomeric C_1_ shifts close to Glu212 (Figure 5). Sidechain rotations are observed in Glu212, Asp214 and Glu217/Gln217 as well as sidechain shifts in the aromatic residues Tyr145 and Trp367 close to Glc−1 and Trp376 above Glc+1. In the product, the glycosidic bond is broken as the proton is transferred to the glycosidic O_4_, while C_1_ migrates closer to Glu212 and the Glc−1 glycosyl ring distorts to a ^*4*^ *C*_*1*_ chair-like conformation (in P1). In this step, the Glc+1 glycosyl ring migrates away from Glc−1 without large conformational change. The geometric parameters for the optimized reactants, TSs, and products for different models can be found in supporting information Supplementary Table S1.

**FIGURE 6:**
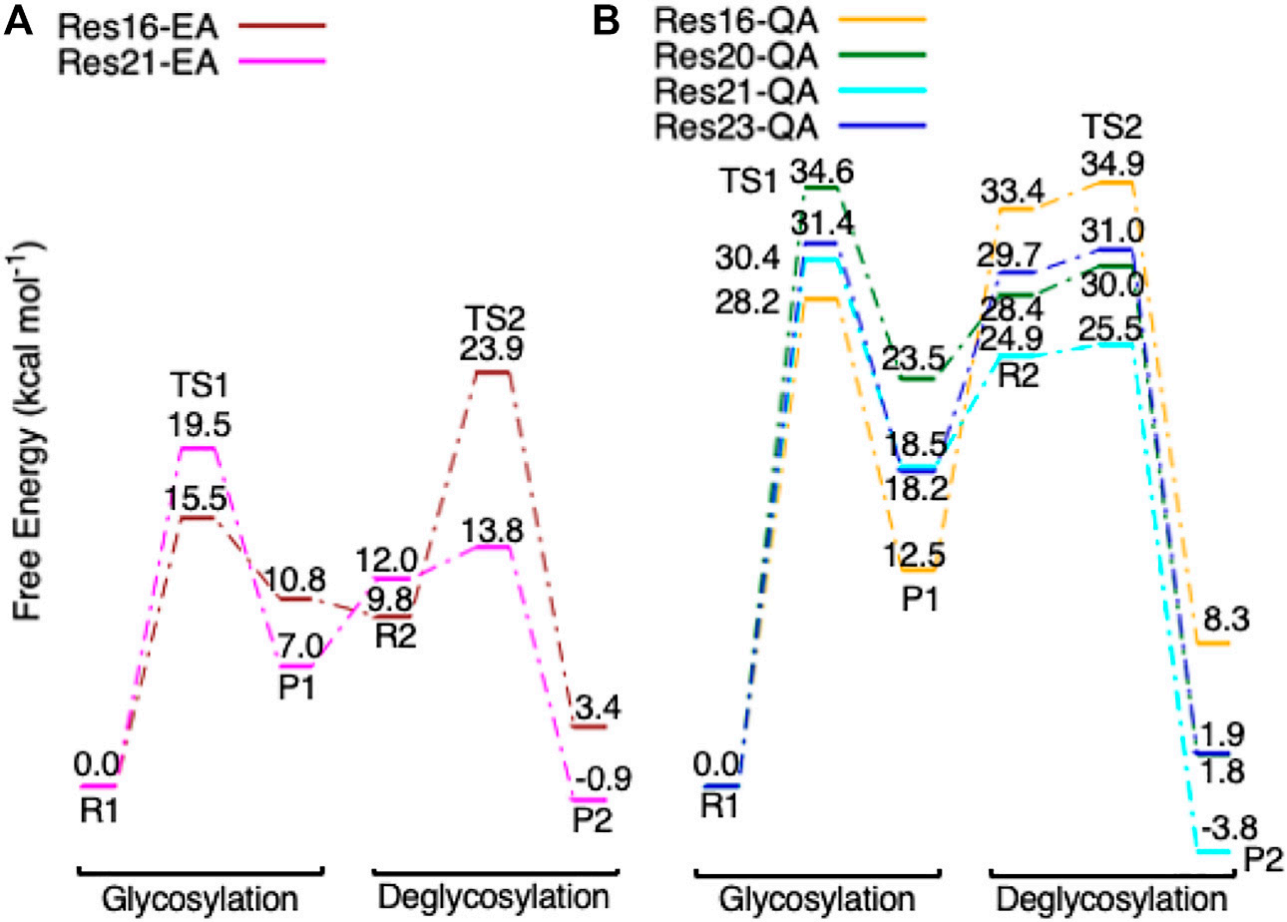
**(A)** Free energy diagram of the glycosylation and deglycosylation for models **Res16-E** (brown) and **Res21-E** (magenta) which are built using PDB 8CEL (wild-type protein), and **(B)**, models **Res16-Q** (orange), **Res20-Q** (green), **Res21-Q** (cyan), and **Res23-Q** (blue) which are built using PDB 4C4C (E217Q, mutated protein).

The free energy diagram for glycosylation is shown in Figure 6. The free energies of activation using **Res16-E** and **Res21-E** models are computed to be 15.5 and 19.5 kcal mol^−1^. Similarly to the earlier computational study using the QM/MM+potential of mean force free energy sampling (15.5 kcal mol^−1^), (Knott et al., 2014b), our free energy of activation in glycosylation using the smaller model is close to the experimentally measured kinetics. The result using the larger model is in excellent agreement with the activation free energy (19.11–19.13 kcal mol^−1^) derived from the experimentally measured rate constants [*k*_*cat*_ = 12.8(±0.2) min^−1^] at 37°C (Ståhlberg et al., 1996). Our two models of 8CEL lead to glycosylation products that are 10.8 and 7.0 kcal mol^−1^ higher in free energy than their corresponding reactants. Using the QM-cluster models based on the E217Q mutated 4C4C X-ray crystal structure, the activation free energies of glycosylation are computed to be 28.2, 34.6, and 30.4 kcal mol^−1^ for **Res16-Q**, **Res20-Q**, and **Res21-Q**, respectively; and 31.4 kcal mol^−1^ using the maximal RINRUS model **Res23-Q**, which is 7 kcal mol^−1^ higher than the free energy (22.68–22.85 kcal mol^−1^) converted from the experimentally measured rate constants [*k*_*cat*_ = 3.5(±0.5) × 10^–2^ min^−1^] (Ståhlberg et al., 1996). Addition of a water molecule plus Thr246 and Arg251 residues causes the Glc+1 ring to shift less from Glc−1 (C_1_-O_4_ bond length is 2.31 Å in **Res23-Q-TS1** versus 2.91 Å in **Res20-Q-TS1**). All models of the E217Q mutated protein led to glycosylation products that are higher in free energy compared to their corresponding reactant. Clearly, the E217Q mutation is much less kinetically or thermodynamically favored for glycosylation. This nicely corresponds to the hypothesis that the E217Q should destabilize the glycosylation transition state.

The predicted free energy of activation for the glycosylation step of the mutated protein is concerningly higher than the experimentally measured kinetics. However, our QM-cluster model kinetics are still more qualitatively reasonable than some previous Cel7A QM/MM-based models. Disagreement between experiment and theory for the E217Q glycosylation activation free energy may be rationalized by frozen atoms exacerbating the already poor proton donating ability of the Gln217 residue. Alternatively, we may not have exhaustively explored glycosyl ring distortions that may provide further transition state stabilization. Lastly, the somewhat coarse level of electronic structure theory used in our QM-cluster models will always cause some deviation from experimental kinetic and thermodynamic values.

Step 2 Deglycosylation

**FIGURE 7:**
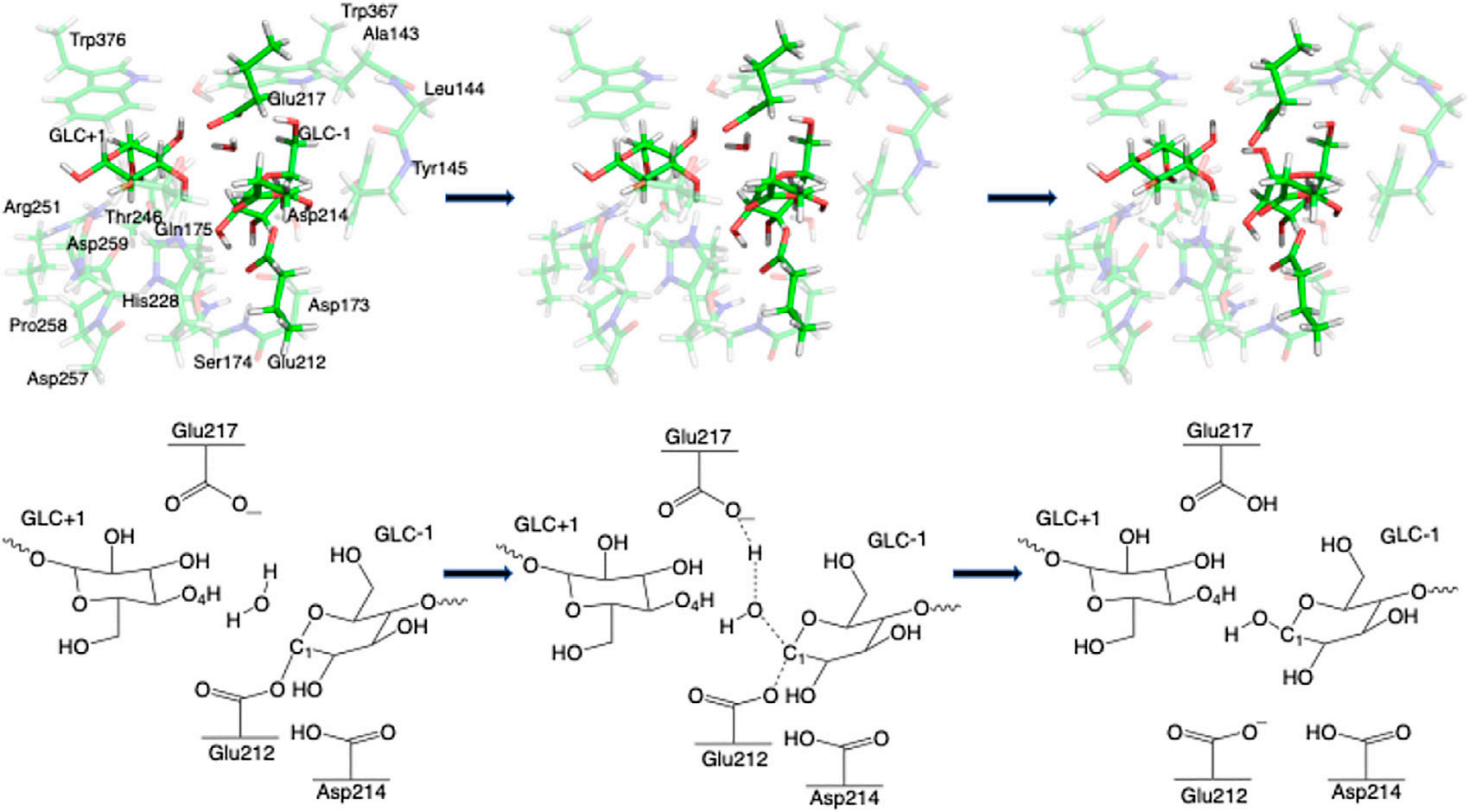
3D and 2D representation of deglycosylation reaction mechanism of the **Res21-E** model of 8CEL.

**FIGURE 8:**
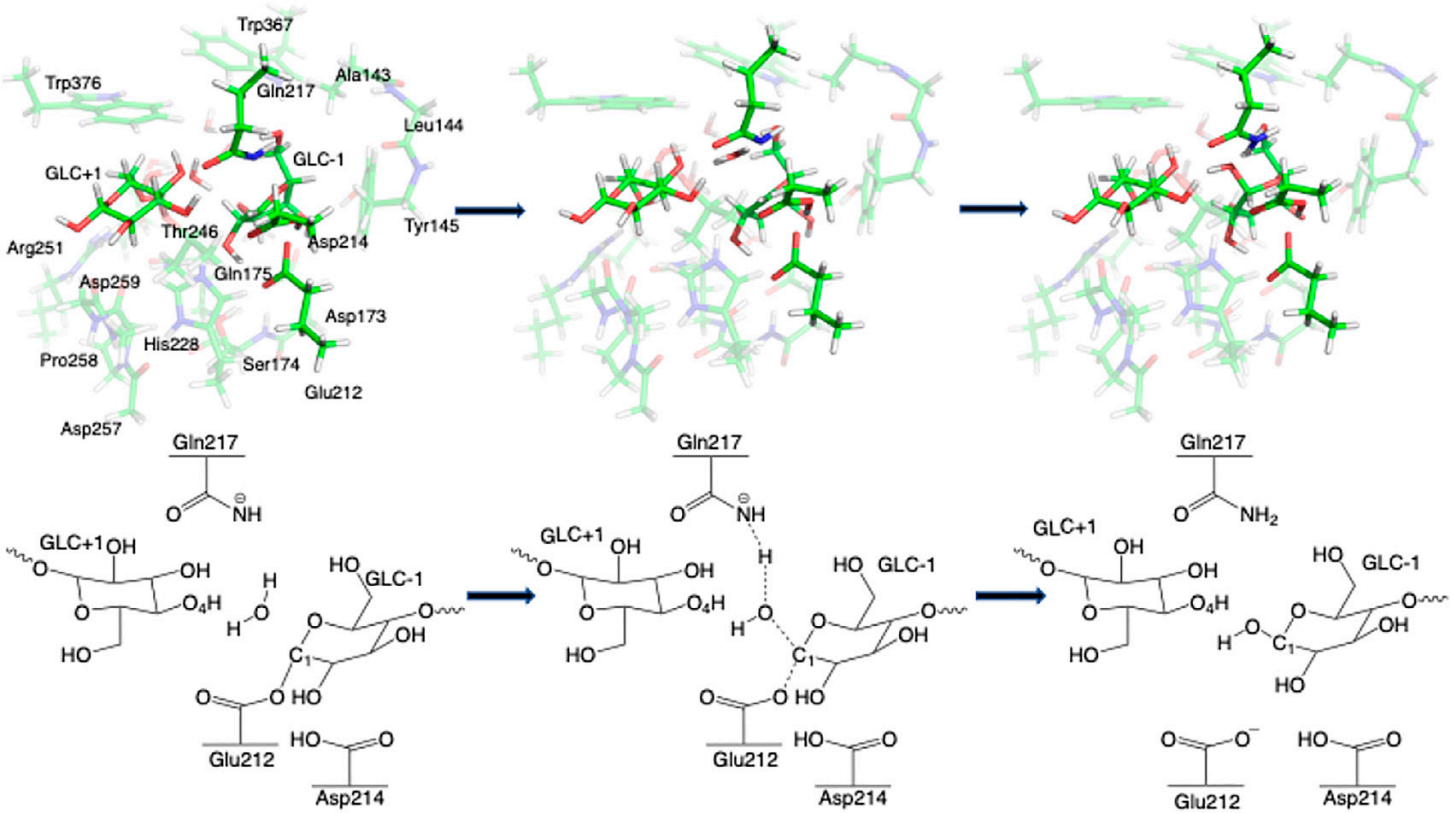
3D and 2D representation of deglycosylation reaction mechanism of the **Res23-Q** model of 4C4C.

**FIGURE 9:**
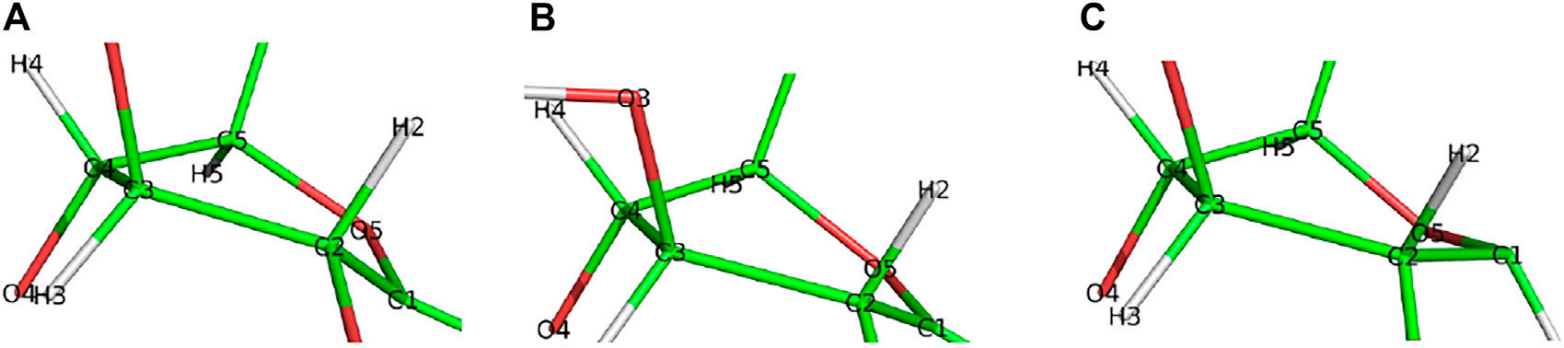
Conformational change of the glycosyl unit Glc−1 in the deglycosylation reaction from **(A)** ^4^C_1_ in R2 to **(B)** ^4^H_3_ in TS2 to **(C)** ^4^E in P2.

One explicit water molecule is added to the active site models for the deglycosylation path, so that the deglycosylation reactant can be connected to the glycosylation product. The representative reactants, transition states (TS), and products of the deglycosylation reaction step are shown in Figures 7, 8, which use models **Res21-E** (3D and 2D representation in Figure 7) and **Res23-Q** (3D and 2D representation in Figure 8). In the reactant (R2), the water positions its oxygen close to the C_1_ of Glc−1, but opposite to Glu212. While one of the hydrogen atoms is close to Glu217-O/Gln217-N, the other hydrogen bonds to the glycosidic O_4_ of Glc+1 and pushes the Glc+1 further away from Glc−1 compared to the glycosylation product. In the TS2, Glc−1 shifts slightly towards the water but further away from Glu212, while the water shifts slightly coupled with sidechain rotation in Glu217/Gln217. A proton transfer from the new water molecule couples with the water nucleophilic attack C_1_ of Glc−1 in the TS2. The proton from the water transfers to Glu217-O/Gln217-N, and the OH group bonds to C_1_ of Glc−1, leading to the glycosyl unit migrating further away from Glu212. The glycosyl unit Glc−1 again undergoes a large conformational change from ^*4*^ *C*_*1*_ to ^*4*^ *H*_*3*_ to ^*4*^ *E* (Figure 9 from R2 to TS2 to P2), which is opposite from the glycosylation process. Most of the residues of the reactant, TS, and product in deglycosylation are structurally unperturbed, while large geometric changes are seen in Trp376 and Trp367, which are near the glycosyl units, and Tyr145, which is close to the Glc−1 glycosyl unit. The geometric parameters for the optimized reactants, TSs, and products of deglycosylation for different QM-cluster models can be found in Supplementary Table S2.

The free energy diagram of deglycosylation is also shown in Figure 6 and Supplementary Table S3. The deglycosylation reactant (R2) is lower in energy than the glycosylation reactant in **Res16-E** model, but higher than the glycosylation reactant in other models. The deglycosylation free energies of activation using the **Res16-E** and **Res21-E** models are computed to be 23.9 and 13.8 kcal mol^−1^ (14.1 and 1.9 kcal mol^−1^ for this elementary step) and the corresponding products are found to be 3.4 kcal mol^−1^ higher and 0.9 kcal mol^−1^ lower in free energy than the glycosylation reactant (R1). The free energies of activation for deglycosylation of the mutated models **Res16-QA**, **Res20-QA**, **Res21-QA** and **Res23-QA** are 1.5, 1.7, 0.7, and 1.3 kcal mol^−1^ for the elementary step, but much higher in overall free energy of activation (34.9, 30.0, 25.5 and 31.0 kcal mol^−1^). Similar to **Res21-EA** model, whose deglycosylation reactant is 5.0 kcal mol^−1^ higher than the glycosylation product plus one water, all E217Q mutated models have deglycosylation reactants 20.9, 4.9, 6.4, and 11.5 kcal mol^−1^ higher in free energy than glycosylation products plus water. Comparing both maximal models, the larger energy difference between the glycosylation product and deglycosylation reactant seen in the mutated model **Res23-QA** may explain the observation of a GEI in the X-ray crystal structure (PDB 4C4C).

Overall, in the RINRUS models of the wild-type protein, the glycosylation transition state is found to be the rate limiting step in our proposed mechanism. While the deglycosylation step in the mutated enzyme has a very high free energy of activation and is competitive with glycosylation, it is still slightly lower in free energy than the TS of the glycosylation step. The glycosylation of the mutant is slower than the wild-type enzyme, which agrees well with experimental observation.

### Alternative Reactant Conformations of the Glycosylation and Deglycosylation Steps

**FIGURE 10:**
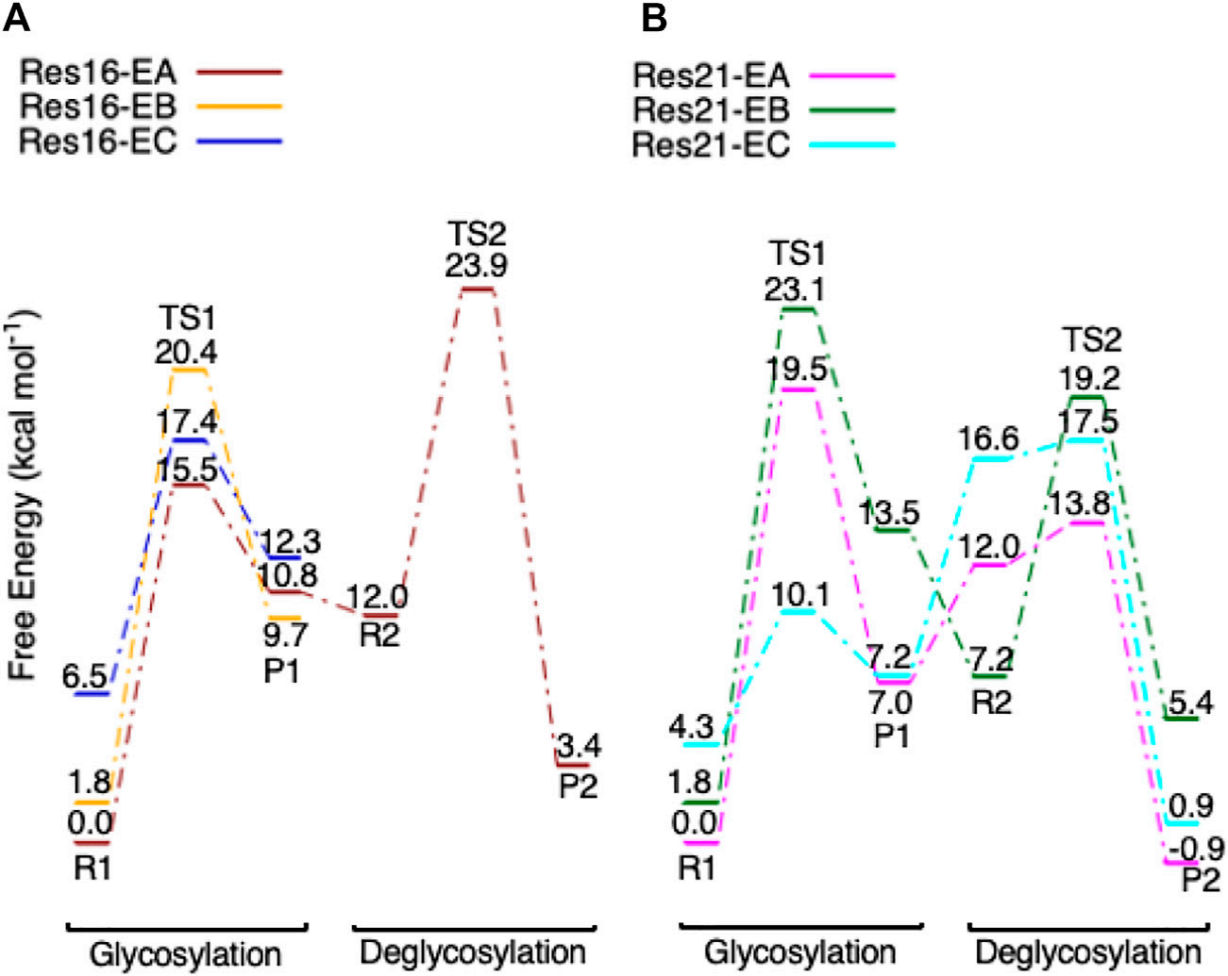
Free energy diagram of glycosylation and deglycosylation for different conformers of models **(A**, left) **Res16-E** and **(B**, right) **Res21-E** based on 8CEL.

For the glycosylation step, using the RINRUS-derived models (**Res16-E and Res21-E**) of the theoretical protein 8CEL enabled us to explore different conformations. Conformational differences (Supplementary Figure S1) are seen in the sidechains of the aromatic residues Tyr145, His228, Trp367, and Trp376 which surround the glycosyl units, Asp214 which hydrogen bonds to the important residue Glu212, and Asp173 and water molecules which form hydrogen bonds with the glycosyl units. The Glu217 sidechain is also quite flexible and can adopt multiple conformations in our QM-cluster models. The free energy diagram of glycosylation using different conformers is shown in Figure 10. Note that the **Res16-EA** and **Res21-EA** models discussed here are shown in Figure 6 and previously discussed. Both **Res16-EB-R1** and **Res21-EB-R1** are slightly higher in energy than **Res16-EA-R1** and **Res21-EA-R1** but lead to a higher energy TS1, which indicates that the glycosylation will be unlikely to take place *via* conformation **B**. The free energy of activation for models **Res16-EC** is slightly higher than that of **Res16-EA**, and reactant **Res16-EC-R1** is 6.5 kcal mol^−1^ higher in free energy than **Res16-EA-R1**, which indicates that the glycosylation reaction will be more likely to occur *via* conformation **A**. While for the maximal model, even though **Res21-EC-R1** is 4.3 kcal mol^−1^ higher in energy than **Res21-EA-R1**, the **Res21-EC** model has a lower free glycosylation activation energy than that of **Res21-EA**. Hence, the deglycosylation was examined and a much higher energy TS was located which indicates that reaction will be unlikely to take place *via* conformation **C**.

**FIGURE 11:**
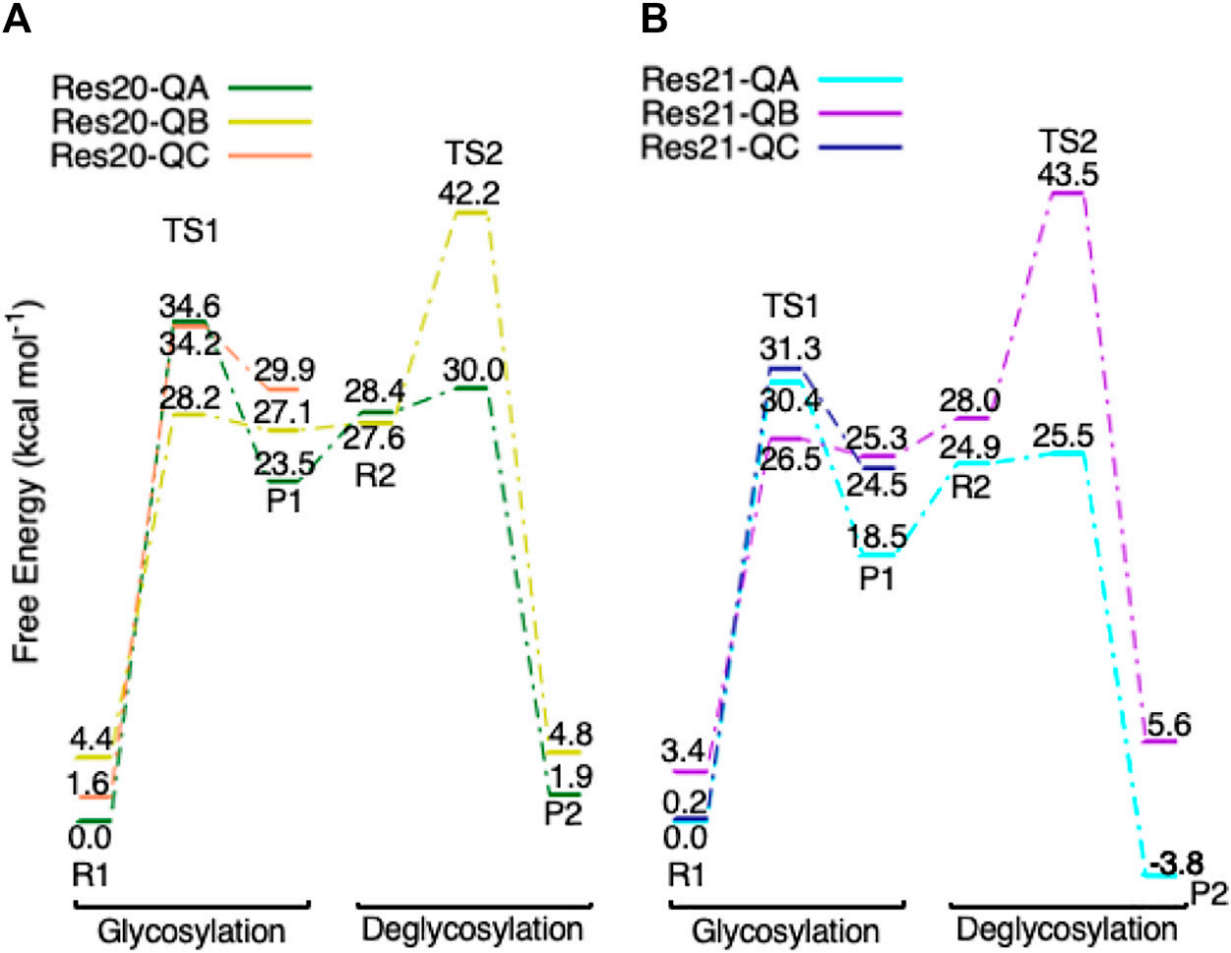
Free energy diagram of glycosylation and deglycosylation for different conformers of models (**A, left**) **Res20-Q** and (**B, right**) **Res21-Q** based on 4C4C.

Three conformations of models **Res20-Q** and **Res21-Q** based on the mutated protein are found in the glycosylation step and the free energy diagram is shown in Figure 11. The glycosylation reactants in the three conformations have different Trp367 and Trp376 sidechain orientations (Supplementary Figures S2B,C); and the reactants (R1) in conformation **B** and **C** are less than 4.5 kcal mol^−1^ higher in free energy than the reactants in conformation **A**. However, the activation free energies of glycosylation of conformation **B** (28.2 and 26.5 kcal mol^−1^ for **Res20-QB** and **Res21-QB**) are lower than those in conformation **A**, but corresponding free energies of activation for deglycosylation were computed to be 40.5 and 43.3 kcal mol^−1^ for **Res20-QB** and **Res21-QB**, respectively, which indicates that even if conformation **B** of the mutated protein is more kinetically favorable for glycosylation, the free energies of activation for deglycosylation are too high to be catalytically viable. These large energy differences in the two steps could be caused by one water shifting to a position close to Glc+1, which blocked Glc+1 from moving away from Glc−1. This water reorientation makes it difficult for the additional water to attack the anomeric carbon. In both **Res20-QC** and **Res21-QC** models, the free energies of TS1s are competitive compared to conformer A, but **Res20-QC-R1** and **Res21-QC-R1** are 1.7 and 0.2 kcal mol^−1^ higher than **Res20-QA-R1** and **Res21-QA-R1** and the glycosylation products (P1) of both conformer **C** are higher in energy than those of conformer **A**, which may lead to a similar deglycosylation reactant in conformer **A**.

**FIGURE 12:**
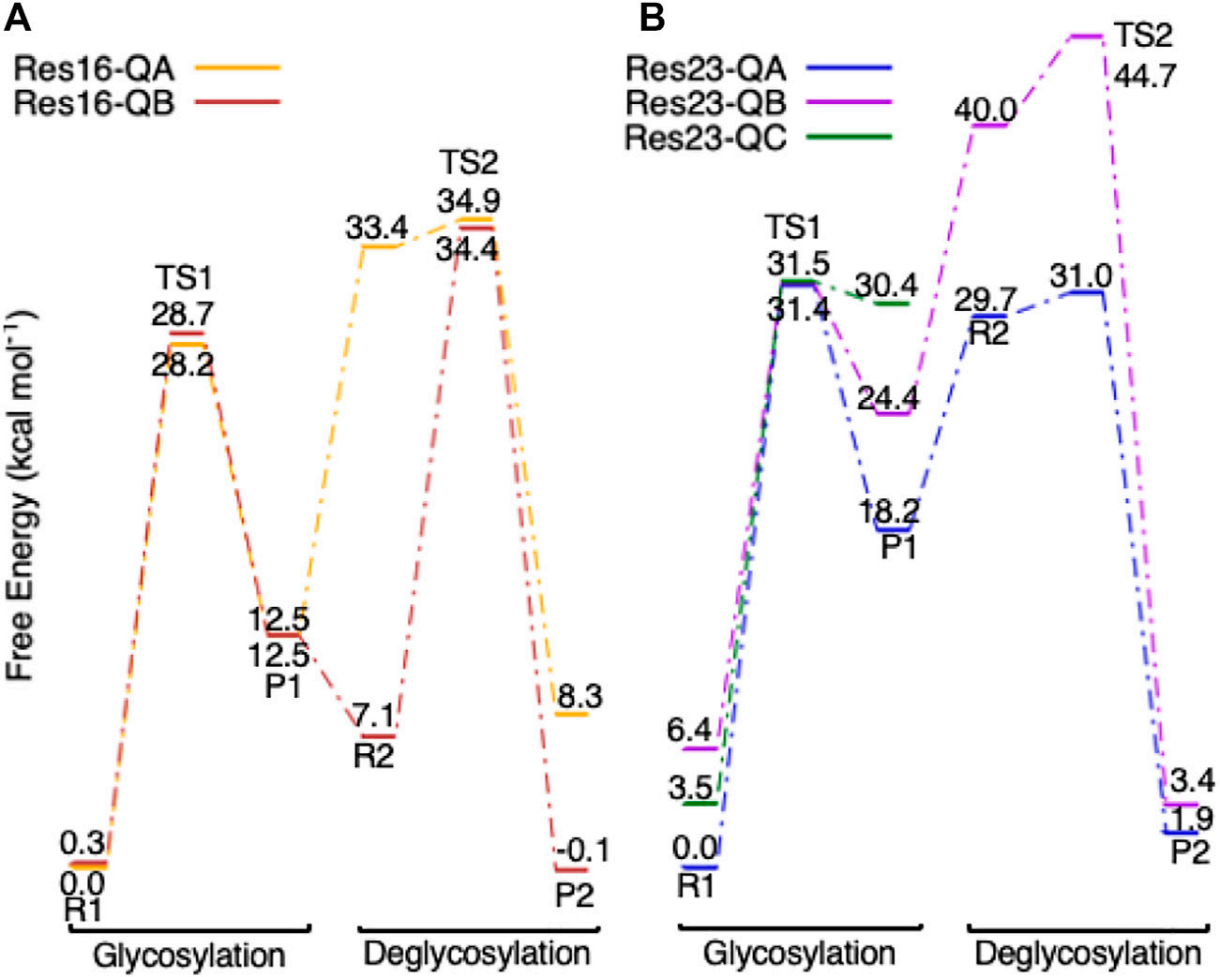
Free energy diagram of glycosylation and deglycosylation for different conformers of models **(A**, left) **Res16-Q** and **(B**, right) **Res23-Q** based on 4C4C.

Multiple conformations of **Res16-Q** and **Res23-Q** are located for both glycosylation and deglycosylation. The free energy diagrams of both models are shown in Figure 12. The reactants, TS, and products in glycosylation step are very similar in **Res16-QA** and **Res16-QB** models; only small differences are seen in Trp367, Trp376 and waters. Both conformers have very high energy deglycosylation TSs, indicating that large QM-cluster models are needed to study the reactions of GHs. Three conformations of the maximal model are examined. Like **Res20-Q** and **Res21-Q,** the TSs in the three additional conformations have almost the same free energy, even though the reactants of **Res23-QB** and **Res23-QC** are 6.4 and 3.5 kcal mol^−1^ higher than **Res23-QA-R1.** For conformer **B** the deglycosylation has a very high energy TS2, indicating the reaction is unlikely to take place *via* this conformation. The product of conformer **C** is also higher in free energy than that of conformer **A**. Overall, conformation **A** is the most energetically favored.

## Conclusion

Cellulose hydrolysis by the cellobiohydrolase 7A (also known as CBHI) enzyme *via* a two-step retaining process is elucidated by two QM-cluster models built on the wild-type theoretical protein structure PDB:8CEL and four models built on the X-ray crystal structure PDB:4C4C in which Glu217 is mutated to Gln. In the glycosylation step, a proton from residue 217 is transferred to the glycosyl unit coupled with the glycosidic bond cleavage. Large conformational change is observed in the Glc−1 unit in both the wild-type and mutated enzyme models. The catalytic trio Glu212, Asp214 and Glu217/Gln217 are important for the reaction. Mutation of Glu to Gln leads to a weaker proton donor that increases the free energy of activation in both glycosylation and deglycosylation. These three residues are also quite flexible as multiple sidechain orientations are seen in the X-ray crystal structure as well as in the multiple pathways proposed in our study. In the deglycosylation step, one explicit water molecule is added to the models and the free energy of activation is lower than those in the glycosylation step in the wild-type protein, indicating glycosylation is the rate-limiting step. The deglycosylation step of the mutated protein model becomes more competitive kinetically. Large QM-cluster models (**Res21-EA**, **Res20-QA**, **Res21-QA**, and **Res23-QA**) lead to a low energy deglycosylation product, hence the full catalytic cycle is thermodynamically favored.

The RINs based on 8CEL and 4C4C are slightly different. However, the fringe residues Arg251 and Thr246, which have fewer interaction counts with the glycosyl units are found to have minor effect on the kinetics of the proposed mechanisms. The free energy of activation of glycosylation is only lowered by 1.5 kcal mol^−1^ when adding Arg251 to **Res20-QA** (to form **Res21-QA)**; the glycosylation activation free energy is lowered by 3.0 kcal mol^−1^ when adding Thr246 and one water to **Res21-QA** (to form **Res23-QA**). All RINRUS-built models provide free energies of activation for the glycosylation step which are in good agreement with experimental measurements of *k*_*cat*_ values for the wild-type protein and its E217Q mutant. Enzymatic reaction studies based on the residue interaction network and QM-cluster models automatically generated by RINRUS can provide good structural and energetic results for better understanding of enzyme reaction mechanisms. The RINRUS workflow is inherently reproducible, which will be greatly beneficial to the computational enzymology community.

This is the first time that RINRUS has been used to explore thermodynamic and kinetic changes of an enzymatic reaction based on point mutation, and RINRUS provides results which are reasonably in good agreement with experimental observation and would be useful to apply to study other metal-free enzyme systems. Based on this study along with our previous QM-cluster studies of different enzymatic reaction, large QM-cluster models with typical 200–300 atoms provide better results than smaller sized models. RINRUS-built QM-cluster models should more accurately predict thermodynamic and kinetic results if conformational sampling is included, which may be necessary when glycosyl units are present in the active site.

## Author Information

Qianyi Cheng – Department of Chemistry, University of Memphis, Memphis, TN, 38152. Nathan J. DeYonker – Department of Chemistry, University of Memphis, Memphis, TN, 38152.

## Data Availability

The original contributions presented in the study are included in the article/Supplementary Materials, further inquiries can be directed to the corresponding authors.

## References

[B2] Barnett C. B. Wilkinson K. A. Naidoo K. J. (2011). Molecular Details from Computational Reaction Dynamics for the Cellobiohydrolase I Glycosylation Reaction. J. Am. Chem. Soc. 133, 19474–19482. 10.1021/ja206842j 22007863

[B3] Barone V. Cossi M. (1998). Quantum Calculation of Molecular Energies and Energy Gradients in Solution by a Conductor Solvent Model. J. Phys. Chem. A. 102, 1995–2001. 10.1021/jp9716997

[B4] Becke A. D. (1993). Density‐functional Thermochemistry. III. The Role of Exact Exchange. J. Chem. Phys. 98, 5648–5652. 10.1063/1.464913

[B5] Blomberg M. R. A. Borowski T. Himo F. Liao R.-Z. Siegbahn P. E. M. (2014). Quantum Chemical Studies of Mechanisms for Metalloenzymes. Chem. Rev. 114, 3601–3658. 10.1021/cr400388t 24410477

[B6] Brás N. F. Moura-Tamames S. A. Fernandes P. A. Ramos M. J. (2008). Mechanistic Studies on the Formation of Glycosidase-Substrate and Glycosidase-Inhibitor Covalent Intermediates. J. Comput. Chem. 29, 2565–2574. 10.1002/jcc.21013 18470964

[B7] Bu L. Beckham G. T. Shirts M. R. Nimlos M. R. Adney W. S. Himmel M. E. (2011). Probing Carbohydrate Product Expulsion from a Processive Cellulase with Multiple Absolute Binding Free Energy Methods. J. Biol. Chem. 286, 18161–18169. 10.1074/jbc.M110.212076 21454590 PMC3093888

[B8] Cantarel B. L. Coutinho P. M. Rancurel C. Bernard T. Lombard V. Henrissat B. (2009). The Carbohydrate-Active EnZymes Database (CAZy): an Expert Resource for Glycogenomics. Nucleic Acids Res. 37, D233–D238. 10.1093/nar/gkn663 18838391 PMC2686590

[B9] Cheng Q. DeYonker N. J. (2020). Acylation and Deacylation Mechanism and Kinetics of Penicillin G Reaction with Streptomyces R61 DD ‐peptidase. J. Comput. Chem. 41, 1685–1697. 10.1002/jcc.26210 32323874

[B10] Cheng Q. DeYonker N. J. (2021). QM-cluster Model Study of the Guaiacol Hydrogen Atom Transfer and Oxygen Rebound with Cytochrome P450 Enzyme GcoA. J. Phys. Chem. B 125, 3296–3306. 10.1021/acs.jpcb.0c10761 33784103

[B11] Chundawat S. P. S. Beckham G. T. Himmel M. E. Dale B. E. (2011). Deconstruction of Lignocellulosic Biomass to Fuels and Chemicals. Annu. Rev. Chem. Biomol. Eng. 2, 121–145. 10.1146/annurev-chembioeng-061010-114205 22432613

[B12] Cossi M. Rega N. Scalmani G. Barone V. (2003). Energies, Structures, and Electronic Properties of Molecules in Solution with the C-PCM Solvation Model. J. Comput. Chem. 24, 669–681. 10.1002/jcc.10189 12666158

[B13] Cremer D. Pople J. A. (1975). General Definition of Ring Puckering Coordinates. J. Am. Chem. Soc. 97, 1354–1358. 10.1021/ja00839a011

[B14] Csermely P. (2008). Creative Elements: Network-Based Predictions of Active Centres in Proteins and Cellular and Social Networks. Trends Biochem. Sci. 33, 569–576. 10.1016/j.tibs.2008.09.006 18945619

[B15] Davies G. J. Wilson K. S. Henrissat B. (1997). Nomenclature for Sugar-Binding Subsites in Glycosyl Hydrolases. Biochem. J. 321, 557–559. 10.1042/bj3210557 9020895 PMC1218105

[B16] Davies G. J. Mackenzie L. Varrot A. Dauter M. Brzozowski A. M. Schülein M. (1998). Snapshots along an Enzymatic Reaction Coordinate: Analysis of a Retaining β-Glycoside Hydrolase,. Biochemistry 37, 11707–11713. 10.1021/bi981315i 9718293

[B17] Davies G. J. Ducros V. M.-A. Varrot A. Zechel D. L. (2003). Mapping the Conformational Itinerary of β-glycosidases by X-ray Crystallography. Biochem. Soc. Trans. 31, 523–527. 10.1042/bst0310523 12773149

[B18] del Sol A. Fujihashi H. Amoros D. Nussinov R. (2006). Residue Centrality, Functionally Important Residues, and Active Site Shape: Analysis of Enzyme and Non-enzyme Families. Protein Sci. 15, 2120–2128. 10.1110/ps.062249106 16882992 PMC2242611

[B19] DeYonker N. J. Webster C. E. (2013). Phosphoryl Transfers of the Phospholipase D Superfamily: A Quantum Mechanical Theoretical Study. J. Am. Chem. Soc. 135, 13764–13774. 10.1021/ja4042753 24007383

[B20] DeYonker N. J. Webster C. E. (2015). A Theoretical Study of Phosphoryl Transfers of Tyrosyl-DNA Phosphodiesterase I (Tdp1) and the Possibility of a "Dead-End" Phosphohistidine Intermediate. Biochemistry 54, 4236–4247. 10.1021/acs.biochem.5b00396 26121557

[B21] Di Paola L. De Ruvo M. Paci P. Santoni D. Giuliani A. (2013). Protein Contact Networks: An Emerging Paradigm in Chemistry. Chem. Rev. 113, 1598–1613. 10.1021/cr3002356 23186336

[B22] Divne C. Ståhlberg J. Reinikainen T. Ruohonen L. Pettersson G. Knowles J. K. C. (1994). The Three-Dimensional Crystal Structure of the Catalytic Core of Cellobiohydrolase I from Trichoderma Reesei. Science 265, 524–528. 10.1126/science.8036495 8036495

[B23] Divne C. Ståhlberg J. Teeri T. T. Jones T. A. (1998). High-resolution crystal Structures Reveal How a Cellulose Chain Is Bound in the 50 Å Long Tunnel of Cellobiohydrolase I from Trichoderma Reesei 1 1Edited by K. Nagai. J. Mol. Biol. 275, 309–325. 10.1006/jmbi.1997.1437 9466911

[B24] Doncheva N. T. Klein K. Domingues F. S. Albrecht M. (2011). Analyzing and Visualizing Residue Networks of Protein Structures. Trends Biochem. Sci. 36, 179–182. 10.1016/j.tibs.2011.01.002 21345680

[B25] Foresman J. B. Frisch Æ. (1996). Exploring Chemistry with Electronic Structure Methods. 2nd ed., 266. Pittsburgh: PA Gaussian Inc., 278–283.

[B26] Frisch M. J. Trucks G. W. Schlegel H. B. Scuseria G. E. Robb M. A. Cheeseman J. R. (2016). Gaussian 16 Revision B.01. Wallingford, CT: Gaussian Inc.

[B27] Fukui K. (1970). Formulation of the Reaction Coordinate. J. Phys. Chem. 74, 4161–4163. 10.1021/j100717a029

[B28] Fukui K. (1981). The Path of Chemical Reactions - the IRC Approach. Acc. Chem. Res. 14, 363–368. 10.1021/ar00072a001

[B29] Grimme S. Antony J. Ehrlich S. Krieg H. (2010). A Consistent and Accurate Ab Initio Parametrization of Density Functional Dispersion Correction (DFT-D) for the 94 Elements H-Pu. J. Chem. Phys. 132, 154104. 10.1063/1.3382344 20423165

[B30] Grimme S. Ehrlich S. Goerigk L. (2011). Effect of the Damping Function in Dispersion Corrected Density Functional Theory. J. Comput. Chem. 32, 1456–1465. 10.1002/jcc.21759 21370243

[B31] Hariharan P. C. Pople J. A. (1973). The Influence of Polarization Functions on Molecular Orbital Hydrogenation Energies. Theoret. Chim. Acta 28, 213–222. 10.1007/BF00533485

[B32] Hehre W. J. Ditchfield R. Pople J. A. (1972). Self-Consistent Molecular Orbital Methods. XII. Further Extensions of Gaussian-type Basis Sets for Use in Molecular Orbital Studies of Organic Molecules. J. Chem. Phys. 56, 2257–2261. 10.1063/1.1677527

[B33] Igarashi K. Uchihashi T. Koivula A. Wada M. Kimura S. Okamoto T. (2011). Traffic Jams Reduce Hydrolytic Efficiency of Cellulase on Cellulose Surface. Science 333, 1279–1282. 10.1126/science.1208386 21885779

[B1] IUPAC-IUB (1980). Conformational Nomenclature for Five and Six-Membered Ring Forms of Monosaccharides and Their Derivatives. Recommendations 1980. Eur. J. Biochem. 111, 295–298. 10.1111/j.1432-1033.1980.tb04941.x 7460897

[B34] Kern M. McGeehan J. E. Streeter S. D. Martin R. N. A. Besser K. Elias L. (2013). Structural Characterization of a Unique marine Animal Family 7 Cellobiohydrolase Suggests a Mechanism of Cellulase Salt Tolerance. Proc. Natl. Acad. Sci. U.S.A. 110, 10189–10194. 10.1073/pnas.1301502110 23733951 PMC3690837

[B35] Klemm D. Heublein B. Fink H.-P. Bohn A. (2005). Cellulose: Fascinating Biopolymer and Sustainable Raw Material. Angew. Chem. Int. Ed. 44, 3358–3393. 10.1002/anie.200460587 15861454

[B36] Knott B. C. Crowley M. F. Himmel M. E. Ståhlberg J. Beckham G. T. (2014a). Carbohydrate-Protein Interactions that Drive Processive Polysaccharide Translocation in Enzymes Revealed from a Computational Study of Cellobiohydrolase Processivity. J. Am. Chem. Soc. 136, 8810–8819. 10.1021/ja504074g 24869982

[B37] Knott B. C. Haddad Momeni M. Crowley M. F. Mackenzie L. F. Götz A. W. Sandgren M. (2014b). The Mechanism of Cellulose Hydrolysis by a Two-step, Retaining Cellobiohydrolase Elucidated by Structural and Transition Path Sampling Studies. J. Am. Chem. Soc. 136, 321–329. 10.1021/ja410291u 24341799

[B38] Knott B. C. Erickson E. Allen M. D. Gado J. E. Graham R. Kearns F. L. (2020). Characterization and Engineering of a Two-Enzyme System for Plastics Depolymerization. Proc. Natl. Acad. Sci. U.S.A. 117, 25476–25485. 10.1073/pnas.2006753117 32989159 PMC7568301

[B39] Koshland D. E. (1953). Stereochemistry and the Mechanism of Enzymatic Reactions. Biol. Rev. 28, 416–436. 10.1111/j.1469-185X.1953.tb01386.x

[B40] Lee C. Yang W. Parr R. G. (1988). Development of the Colle-Salvetti Correlation-Energy Formula into a Functional of the Electron Density. Phys. Rev. B 37, 785–789. 10.1103/PhysRevB.37.785 9944570

[B41] Li J. Du L. Wang L. (2010). Glycosidic-Bond Hydrolysis Mechanism Catalyzed by Cellulase Cel7A from Trichoderma Reesei: A Comprehensive Theoretical Study by Performing MD, QM, and QM/MM Calculations. J. Phys. Chem. B 114, 15261–15268. 10.1021/jp1064177 21028861

[B42] Mackenzie L. F. Sulzenbacher G. Divne C. Jones T. A. Wöldike H. F. Schülein M. (1998). Crystal Structure of the Family 7 Endoglucanase I (Cel7B) from Humicola Insolens at 2.2 Å Resolution and Identification of the Catalytic Nucleophile by Trapping of the Covalent Glycosyl-Enzyme Intermediate. Biochem. J. 335, 409–416. 10.1042/bj3350409 9761741 PMC1219796

[B43] Martinez D. Berka R. M. Henrissat B. Saloheimo M. Arvas M. Baker S. E. (2008). Genome Sequencing and Analysis of the Biomass-Degrading Fungus Trichoderma Reesei (Syn. Hypocrea Jecorina). Nat. Biotechnol. 26, 553–560. 10.1038/nbt1403 18454138

[B44] Mayes H. B. Broadbelt L. J. Beckham G. T. (2014). How Sugars Pucker: Electronic Structure Calculations Map the Kinetic Landscape of Five Biologically Paramount Monosaccharides and Their Implications for Enzymatic Catalysis. J. Am. Chem. Soc. 136, 1008–1022. 10.1021/ja410264d 24368073

[B45] Pan X.-L. Liu W. Liu J.-Y. (2013). Mechanism of the Glycosylation Step Catalyzed by Human α-Galactosidase: A QM/MM Metadynamics Study. J. Phys. Chem. B 117, 484–489. 10.1021/jp308747c 23249437

[B46] Petersson G. A. Al‐Laham M. A. (1991). A Complete Basis Set Model Chemistry. II. Open‐shell Systems and the Total Energies of the First‐row Atoms. J. Chem. Phys. 94, 6081–6090. 10.1063/1.460447

[B47] Satoh H. Manabe S. (2013). Design of Chemical Glycosyl Donors: Does Changing Ring Conformation Influence Selectivity/reactivity? Chem. Soc. Rev. 42, 4297. 10.1039/c3cs35457a 23364773

[B48] Shannon P. Markiel A. Ozier O. Baliga N. S. Wang J. T. Ramage D. (2003). Cytoscape: A Software Environment for Integrated Models of Biomolecular Interaction Networks. Genome Res. 13, 2498–2504. 10.1101/gr.1239303 14597658 PMC403769

[B49] Siegbahn P. E. M. Blomberg M. R. A. (2000). Transition-Metal Systems in Biochemistry Studied by High-Accuracy Quantum Chemical Methods. Chem. Rev. 100, 421–438. 10.1021/cr980390w 11749242

[B50] Ståhlberg J. Divne C. Koivula A. Piens K. Claeyssens M. Teeri T. T. (1996). Activity Studies and Crystal Structures of Catalytically Deficient Mutants of Cellobiohydrolase I fromTrichoderma Reesei. J. Mol. Biol. 264, 337–349. 10.1006/jmbi.1996.0644 8951380

[B51] Sulzenbacher G. Schülein M. Davies G. J. (1997). Structure of the Endoglucanase I from Fusarium Oxysporum: Native, Cellobiose, and 3,4-Epoxybutyl β-d-Cellobioside-Inhibited Forms, at 2.3 Å Resolution. Biochemistry 36, 5902–5911. 10.1021/BI962963+ 9153432

[B52] Summers T. J. Cheng Q. DeYonker N. J. (2018). A Transition State "trapped"? QM-Cluster Models of Engineered Threonyl-tRNA Synthetase. Org. Biomol. Chem. 16, 4090–4100. 10.1039/C8OB00540K 29671451

[B53] Summers T. J. Daniel B. P. Cheng Q. DeYonker N. J. (2019). Quantifying Inter-residue Contacts through Interaction Energies. J. Chem. Inf. Model. 59, 5034–5044. 10.1021/acs.jcim.9b00804 31756092

[B54] Summers T. J. Cheng Q. Palma M. A. Pham D.-T. Kelso D. K. Webster C. E. (2021). Cheminformatic Quantum Mechanical Enzyme Model Design: A Catechol-O-Methyltransferase Case Study. Biophysical J. 120, 3577–3587. 10.1016/j.bpj.2021.07.029 PMC8456309 34358526

[B55] Taylor L. E. Knott B. C. Baker J. O. Alahuhta P. M. Hobdey S. E. Linger J. G. (2018). Engineering Enhanced Cellobiohydrolase Activity. Nat. Commun. 9, 1186. 10.1038/s41467-018-03501-8 29567941 PMC5864845

[B56] Updegraff D. M. (1969). Semimicro Determination of Cellulose Inbiological Materials. Anal. Biochem. 32, 420–424. 10.1016/S0003-2697(69)80009-6 5361396

[B57] Vermaas J. V. Crowley M. F. Beckham G. T. Payne C. M. (2015). Effects of Lytic Polysaccharide Monooxygenase Oxidation on Cellulose Structure and Binding of Oxidized Cellulose Oligomers to Cellulases. J. Phys. Chem. B 119, 6129–6143. 10.1021/acs.jpcb.5b00778 25785779

[B58] Vishveshwara S. Ghosh A. Hansia P. (2009). Intra and Inter-molecular Communications through Protein Structure Network. Cpps 10, 146–160. 10.2174/138920309787847590 19355982

[B59] Vocadlo D. J. Davies G. J. (2008). Mechanistic Insights into Glycosidase Chemistry. Curr. Opin. Chem. Biol. 12, 539–555. 10.1016/j.cbpa.2008.05.010 18558099

[B60] Wolfenden R. Lu X. Young G. (1998). Spontaneous Hydrolysis of Glycosides. J. Am. Chem. Soc. 120, 6814–6815. 10.1021/ja9813055

[B61] Word J. M. Lovell S. C. LaBean T. H. Taylor H. C. Zalis M. E. Presley B. K. (1999a). Visualizing and Quantifying Molecular Goodness-Of-Fit: Small-Probe Contact Dots with Explicit Hydrogen Atoms 1 1Edited by J. Thornton. J. Mol. Biol. 285, 1711–1733. 10.1006/jmbi.1998.2400 9917407

[B62] Word J. M. Lovell S. C. Richardson J. S. Richardson D. C. (1999b). Asparagine and Glutamine: Using Hydrogen Atom Contacts in the Choice of Side-Chain Amide Orientation 1 1Edited by J. Thornton. J. Mol. Biol. 285, 1735–1747. 10.1006/jmbi.1998.2401 9917408

[B63] Yan S. Li T. Yao L. (2011). Mutational Effects on the Catalytic Mechanism of Cellobiohydrolase I from Trichoderma Reesei. J. Phys. Chem. B 115, 4982–4989. 10.1021/jp200384m 21476560

