# Supplementary material for: A Case Study of the Glycoside Hydrolase Enzyme Mechanism Using an Automated QM-Cluster Model Building Toolkit
Source: Front Chem. 2022 Mar 24;10:854318. doi: 10.3389/fchem.2022.854318 (PMC8987026; doi:10.3389/fchem.2022.854318)
Supplement: Supplementary file 2 [file DataSheet1.docx]

**A Case Study of the Glycoside Hydrolase Enzyme Mechanism Using an Automated QM-cluster Model Building Toolkit**

*Qianyi Cheng* and Nathan J. DeYonker**

Department of Chemistry, University of Memphis, Memphis, TN 38152, U.S.A.

E-mail: [qcheng1@memphis.edu](mailto:qcheng1@memphis.edu)*, [ndyonker@memphis.edu](mailto:ndyonker@memphis.edu*)*

Table S1: Bond distances of the optimized reactants, transition states, and products of different conformation of **Res16-E** and **Res21-E** models which are built using PDB 8CEL (E217, wild type protein), and **Res16-Q**, **Res20-Q**, **Res21-Q** and **Res23-Q** which are built using PDB 4C4C (Q217, mutated protein) in glycosylation.

|  |  |  |  |  |  |  |
| --- | --- | --- | --- | --- | --- | --- |
|  | *r*(H_217_-O/N_217_) | *r*(H_217_-O_4_) | *r*(C_4_-O_4_) | *r*(O_4_-C_1_) | *r*(C_1_-O_212_) | *r*(O_212_-H_214_) |
|  |  |  |  |  |  |  |
| R16-EA-R1 | 0.99 | 1.88 | 1.45 | 1.45 | 3.36 | 1.50 |
| R16-EA-TS1 | 1.31 | 1.15 | 1.46 | 1.83 | 2.85 | 1.55 |
| R16-EA-P1 | 1.76 | 1.00 | 1.43 | 3.26 | 2.08 | 1.67 |
|  |  |  |  |  |  |  |
| R16-EB-R1 | 0.99 | 1.79 | 1.45 | 1.46 | 3.48 | 1.41 |
| R16-EB-TS1 | 1.35 | 1.13 | 1.45 | 1.88 | 2.98 | 1.50 |
| R16-EB-P1 | 1.83 | 0.99 | 1.43 | 3.81 | 1.53 | 3.65 |
|  |  |  |  |  |  |  |
| R16-EC-R1 | 0.99 | 1.83 | 1.45 | 1.47 | 3.26 | 1.44 |
| R16-EC-TS1 | 1.34 | 1.12 | 1.46 | 1.84 | 2.93 | 1.50 |
| R16-EC-P1 | 1.77 | 1.00 | 1.43 | 3.32 | 2.00 | 1.65 |
|  |  |  |  |  |  |  |
| R16-QA-R1 | 1.02 | 1.89 | 1.45 | 1.46 | 3.44 | 1.52 |
| R16-QA-TS1 | 1.49 | 1.09 | 1.44 | 2.07 | 2.75 | 1.59 |
| R16-QA-P1 | 1.84 | 1.00 | 1.44 | 4.03 | 1.51 | 2.86 |
|  |  |  |  |  |  |  |
| R16-QB-R1 | 1.02 | 1.89 | 1.45 | 1.46 | 3.42 | 1.52 |
| R16-QB-TS1 | 1.52 | 1.07 | 1.44 | 2.11 | 2.71 | 1.59 |
| R16-QB-P1 | 1.84 | 1.00 | 1.44 | 4.03 | 2.86 | 1.51 |
|  |  |  |  |  |  |  |
| R20-QA-R1 | 1.02 | 1.83 | 1.44 | 1.44 | 3.36 | 1.57 |
| R20-QA-TS1 | 1.73 | 1.01 | 1.43 | 2.64 | 2.24 | 1.70 |
| R20-QA-P1 | 1.77 | 1.01 | 1.45 | 4.08 | 1.53 | 3.03 |
|  |  |  |  |  |  |  |
| R20-QB-R1 | 1.03 | 1.85 | 1.45 | 1.48 | 3.08 | 1.58 |
| R20-QB-TS1 | 1.72 | 1.01 | 1.43 | 2.56 | 2.25 | 1.69 |
| R20-QB-P1 | 1.76 | 1.00 | 1.43 | 2.92 | 1.96 | 1.78 |
|  |  |  |  |  |  |  |
| R20-QC-R1 | 1.02 | 1.85 | 1.45 | 1.45 | 3.19 | 1.56 |
| R20-QC-TS1 | 1.78 | 1.00 | 1.43 | 2.91 | 2.05 | 1.68 |
| R20-QC-P1 | 1.84 | 0.99 | 1.43 | 3.59 | 1.53 | 2.73 |
|  |  |  |  |  |  |  |
| R21-QA-R1 | 1.02 | 1.87 | 1.45 | 1.45 | 3.41 | 1.58 |
| R21-QA-TS1 | 1.66 | 1.03 | 1.44 | 2.33 | 2.52 | 1.65 |
| R21-QA-P1 | 1.74 | 1.02 | 1.44 | 3.65 | 2.00 | 1.72 |
|  |  |  |  |  |  |  |
| R21-QB-R1 | 1.02 | 1.86 | 1.45 | 1.48 | 3.04 | 1.59 |
| R21-QB-TS1 | 1.69 | 1.01 | 1.43 | 2.53 | 2.24 | 1.69 |
| R21-QB-P1 | 1.73 | 1.00 | 1.43 | 2.86 | 1.98 | 1.77 |
|  |  |  |  |  |  |  |
| R21-QC-R1 | 1.02 | 1.88 | 1.45 | 1.45 | 3.42 | 1.58 |
| R21-QC-TS1 | 1.70 | 1.02 | 1.44 | 2.42 | 2.46 | 1.66 |
| R21-QC-P1 | 1.89 | 1.00 | 1.44 | 3.46 | 2.00 | 1.75 |
|  |  |  |  |  |  |  |
| R21-EA-R1 | 0.99 | 1.80 | 1.46 | 1.46 | 3.55 | 1.56 |
| R21-EA-TS1 | 1.39 | 1.13 | 1.48 | 1.74 | 3.08 | 1.64 |
| R21-EA-P1 | 1.81 | 0.98 | 1.44 | 3.25 | 2.17 | 1.62 |
|  |  |  |  |  |  |  |
| R21-EB-R1 | 0.99 | 1.80 | 1.46 | 1.46 | 3.55 | 1.56 |
| R21-EB-TS1 | 1.36 | 1.13 | 1.48 | 1.74 | 3.08 | 1.64 |
| R21-EB-P1 | 1.87 | 0.98 | 1.44 | 3.25 | 2.17 | 1.62 |
|  |  |  |  |  |  |  |
| R21-EC-R1 | 1.00 | 1.68 | 1.45 | 1.50 | 3.14 | 1.53 |
| R21-EC-TS1 | 1.37 | 1.10 | 1.46 | 1.78 | 2.95 | 1.57 |
| R21-EC-P1 | 1.67 | 1.00 | 1.43 | 2.65 | 2.36 | 1.60 |
|  |  |  |  |  |  |  |
| R23-QA-R1 | 1.02 | 1.93 | 1.45 | 1.45 | 3.43 | 1.57 |
| R23-QA-TS1 | 1.71 | 1.02 | 1.44 | 2.45 | 2.48 | 1.65 |
| R23-QA-P1 | 1.77 | 1.01 | 1.44 | 3.67 | 1.94 | 1.78 |
|  |  |  |  |  |  |  |
| R23-QB-R1 | 1.03 | 1.85 | 1.45 | 1.47 | 3.01 | 1.59 |
| R23-QB-TS1 | 1.70 | 1.01 | 1.43 | 2.65 | 2.11 | 1.72 |
| R23-QB-P1 | 1.77 | 1.00 | 1.42 | 3.25 | 2.74 | 1.50 |
|  |  |  |  |  |  |  |
| R23-QC-R1 | 1.02 | 1.89 | 1.45 | 1.45 | 3.39 | 1.57 |
| R23-QC-TS1 | 1.67 | 1.02 | 1.44 | 2.31 | 2.55 | 1.65 |
| R23-QC-P1 | 1.79 | 1.00 | 1.43 | 2.94 | 2.15 | 1.72 |

Table S2: Bond distances of the optimized reactants, transition states, and products of **Res16-EA** and **Res21-EA** models which are built using PDB 8CEL (E217, wild type protein), and **Res16-Q**, **Res20-Q**, **Res21-Q** and **Res23-Q** which are built using PDB 4C4C (Q217, mutated protein) in deglycosylation.

|  |  |  |  |  |  |
| --- | --- | --- | --- | --- | --- |
|  | *r*(H_wat_-O_4_) | *r*(H_wat_-O/N_217_) | *r*(H_wat_-O_wat_) | *r*(O_wat_-C_1_) | *r*(C_1_-O_212_) |
|  |  |  |  |  |  |
| R16-EA-R2 | 2.78 | 1.59 | 1.00 | 3.52 | 1.49 |
| R16-EA-TS2 | 1.72 | 1.53 | 1.01 | 2.17 | 2.57 |
| R16-EA-P2 | 1.74 | 0.99 | 2.61 | 1.45 | 4.38 |
|  |  |  |  |  |  |
| R16-QA-R2 | 1.74 | 2.94 | 0.99 | 3.71 | 1.50 |
| R16-QA-TS2 | 1.68 | 1.65 | 1.01 | 2.33 | 2.50 |
| R16-QA-P2 | 1.76 | 1.01 | 2.90 | 1.45 | 3.79 |
|  |  |  |  |  |  |
| R16-QB-R2 | 1.85 | 1.57 | 1.03 | 3.42 | 1.92 |
| R16-QB-TS2 | 1.71 | 1.52 | 1.04 | 2.36 | 2.24 |
| R16-QB-P2 | 1.66 | 1.03 | 1.71 | 2.08 | 2.79 |
|  |  |  |  |  |  |
| R20-QA-R2 | 1.67 | 1.51 | 1.05 | 2.70 | 2.12 |
| R20-QA-TS2 | 1.66 | 1.49 | 1.06 | 2.55 | 2.19 |
| R20-QA-P2 | 1.62 | 1.02 | 1.78 | 1.47 | 2.84 |
|  |  |  |  |  |  |
| R20-QB-R2 | 1.65 | 2.90 | 0.97 | 3.40 | 1.46 |
| R20-QB-TS2 | 1.79 | 1.65 | 1.01 | 2.24 | 2.43 |
| R20-QB-P2 | 1.69 | 1.01 | 2.52 | 1.46 | 3.36 |
|  |  |  |  |  |  |
| R21-QA-R2 | 1.73 | 1.49 | 1.06 | 3.13 | 2.03 |
| R21-QA-TS2 | 1.67 | 1.48 | 1.06 | 2.55 | 2.18 |
| R21-QA-P2 | 1.62 | 1.02 | 1.79 | 1.47 | 2.82 |
|  |  |  |  |  |  |
| R21-QB-R2 | 1.66 | 2.94 | 0.97 | 3.41 | 1.47 |
| R21-QB-TS2 | 1.79 | 1.67 | 1.01 | 2.19 | 2.45 |
| R21-QB-P2 | 1.71 | 1.01 | 2.61 | 1.45 | 3.29 |
|  |  |  |  |  |  |
| R21-EA-R2 | 1.72 | 1.88 | 0.98 | 3.00 | 2.15 |
| R21-EA-TS2 | 1.69 | 1.75 | 0.98 | 2.48 | 2.44 |
| R21-EA-P2 | 1.65 | 1.04 | 1.49 | 1.46 | 3.10 |
|  |  |  |  |  |  |
| R21-EB-R2 | 1.81 | 1.86 | 0.98 | 3.05 | 2.61 |
| R21-EB-TS2 | 1.81 | 1.85 | 0.98 | 2.88 | 2.70 |
| R21-EB-P2 | 1.64 | 1.03 | 1.52 | 1.45 | 3.91 |
|  |  |  |  |  |  |
| R21-EC-R2 | 1.71 | 1.73 | 0.98 | 2.92 | 2.25 |
| R21-EC-TS2 | 1.71 | 1.72 | 0.98 | 2.86 | 2.28 |
| R21-EC-INT2 | 1.55 | 1.42 | 1.06 | 1.56 | 3.11 |
| R21-EC-TS3 | 1.62 | 1.20 | 1.21 | 1.51 | 3.13 |
| R21-EC-P3 | 1.67 | 1.07 | 1.38 | 1.48 | 3.15 |
|  |  |  |  |  |  |
| R23-QA-R2 | 1.74 | 1.49 | 1.06 | 3.28 | 2.00 |
| R23-QA-TS2 | 1.65 | 1.46 | 1.07 | 2.44 | 2.22 |
| R23-QA-P2 | 1.61 | 1.02 | 1.78 | 1.47 | 2.79 |
|  |  |  |  |  |  |
| R23-QB-R2 | 2.66 | 1.74 | 1.00 | 3.07 | 1.97 |
| R23-QB-TS2 | 2.13 | 1.53 | 1.05 | 2.04 | 2.81 |
| R23-QB-P2 | 2.78 | 1.02 | 3.44 | 1.47 | 3.43 |

Table S3. Reaction free energies (kcal mol^-1^) of the optimized reactants, transition states, and products of different conformation of **Res16-E** and **Res21-E** models which are built using PDB 8CEL (E217, wild type protein), and **Res16-Q**, two conformations of **Res20-Q** and **Res21-Q** as well as the maximal model **Res23-Q** which are built using PDB 4C4C (Q217, mutated protein) in glycosylation and deglycosylation. In the deglycosylation, one water is added to the model. The free energy differences between R2 and R1 plus one water are computed as the relative free energy differences. The relative free energies for the deglycosylation elementary step are shown in parenthesis.

|  |  | |  | |  |  |  | |  |  |
| --- | --- | --- | --- | --- | --- | --- | --- | --- | --- | --- |
|  |  | Glycosylation |  | | | Deglycosylation | | | | |
|  | R1 | TS1 | | P1 | | R2 | | TS2 | | P2 |
| Res16-EA | 0.00 | 15.46 | | 10.81 | | 9.97 | | 23.88 | | 3.38 |
|  |  |  | |  | | (0.00) | | (14.09) | | (-6.40) |
| Res16-EB | 1.80 | 20.39 | | 9.71 | |  | |  | |  |
|  |  |  | |  | |  | |  | |  |
| Res16-EC | 6.48 | 17.38 | | 12.34 | |  | |  | |  |
|  |  |  | |  | |  | |  | |  |
| Res21-EA | 0.00 | 19.54 | | 6.95 | | 11.97 | | 13.83 | | -0.86 |
|  |  |  | |  | | (0.00) | | (1.86) | | (-12.83) |
| Res21-EB | 1.78 | 23.04 | | 13.47 | | 7.23 | | 19.24 | | 5.37 |
|  |  |  | |  | | (0.00) | | (12.01) | | (-1.85) |
| Res21-EC | 4.30 | 10.08 | | 7.24 | | 16.62 | | 17.45 | | 0.88 |
|  |  |  | |  | | (0.00) | | (0.83) | | (-15.74) |
|  |  |  | |  | |  | |  | |  |
| Res16-QA | 0.00 | 28.15 | | 12.51 | | 33.39 | | 34.91 | | 8.26 |
|  |  |  | |  | | (0.00) | | (1.53) | | (-25.13) |
| Res16-QB | 0.28 | 28.73 | | 12.53 | | 7.09 | | 34.39 | | -0.14 |
|  |  |  | |  | | (0.00) | | (29.41) | | (-5.93) |
| Res20-QA | 0.00 | 32.60 | | 28.24 | | 26.71 | | 28.38 | | 0.16 |
|  |  |  | |  | | (0.00) | | (1.67) | | (-26.55) |
| Res20-QB | 2.78 | 26.53 | | 25.40 | | 25.91 | | 40.54 | | 3.14 |
|  |  |  | |  | | (0.00) | | (14.64) | | (-22.77) |
| Res21-QA | 0.00 | 31.12 | | 24.27 | | 24.66 | | 25.31 | | -4.02 |
|  |  |  | |  | | (0.00) | | (0.65) | | (-28.68) |
| Res21-QB | 3.22 | 26.32 | | 25.15 | | 27.76 | | 43.32 | | 5.36 |
|  |  |  | |  | | (0.00) | | (15.55) | | (-22.40) |
| Res23-QA | 0.00 | 31.38 | | 18.18 | | 29.71 | | 30.98 | | 1.85 |
|  |  |  |  | | | (0.00) | | (1.27) | | (-27.85) |
| Res23-QB | 6.41 | 31.49 | | 24.42 | | 39.96 | | 44.73 | | 3.37 |
|  |  |  |  | | | (0.00) | | (4.77) | | (-36.59) |
| Res23-QC | 3.49 | 31.54 | | 30.37 | |  | |  | |  |
|  |  |  |  | | |  | |  | |  |


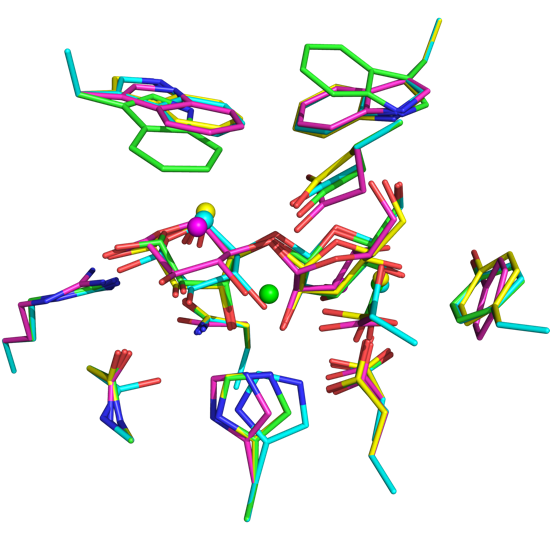

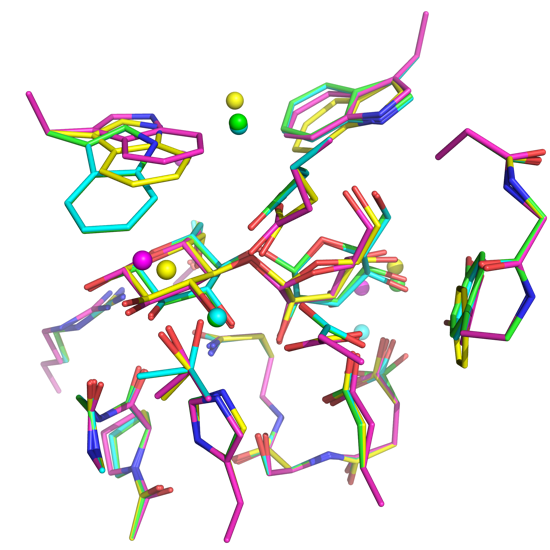


W376

W367

Y145

D173

Q175

D214

E212

H228

R251

E217

W376

W367

A143

L144

Y145

D173

S174

Q175

D214

E212

H228

D257

P258

D259

R251

T246

T226

E217

D259

b.

a.

Figure S1. Aligned optimized (a) **Res16-EA-R1** and (b) **Res21-EA-R1** (green) to the model of X-ray crystal structure (magenta). Waters are shown in spheres. The higher energy conformers **B** and **C** of **Res16-E** and **Res21-E** reactants of 8CEL are shown in cyan and yellow.


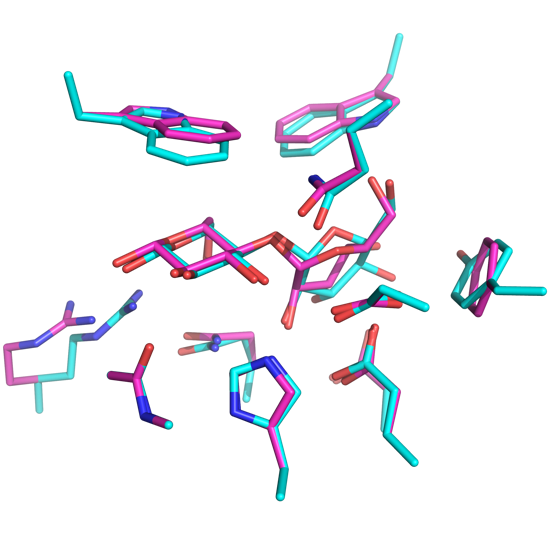

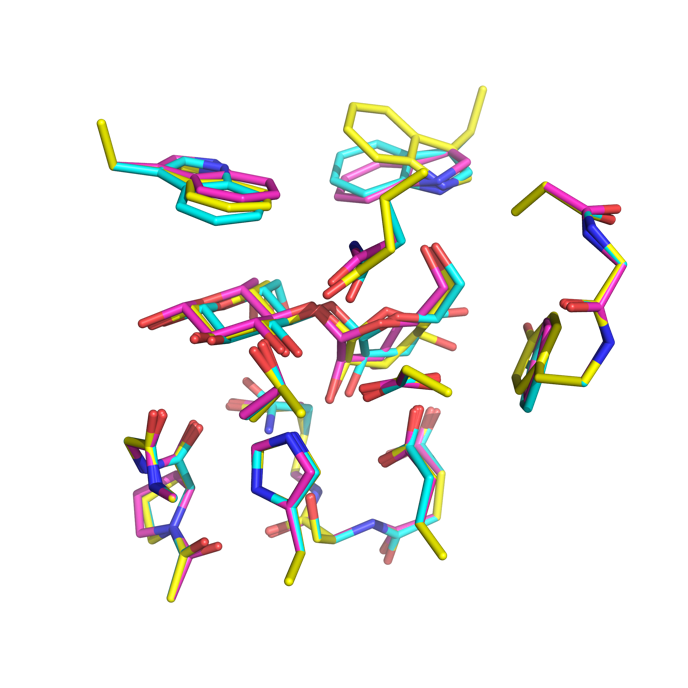


W376

W367

A143

L144

Y145

D173

S174

Q175

D214

E212

H228

D257

P258

D259

T226

Q217

W376

W367

Y145

D173

Q175

D214

E212

H228

D259

R251

Q217

b.

a.


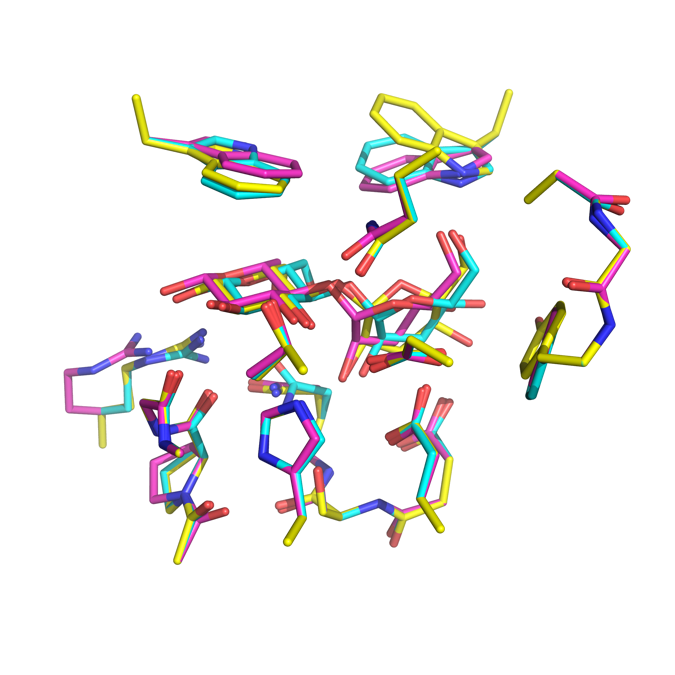

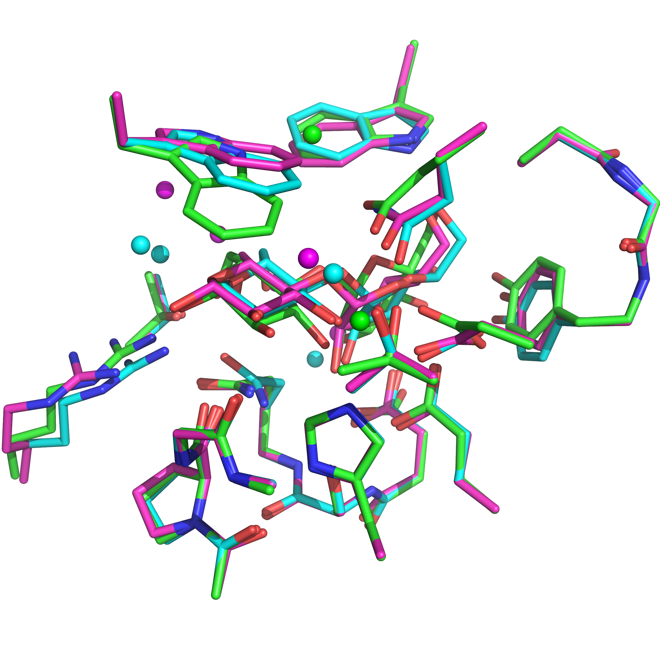


W376

W367

A143

L144

Y145

D173

Q175

D214

E212

H228

D257

P258

D259

R251

T226

Q217

S174

c.

d.

T246

W376

W367

A143

L144

Y145

D173

S174

Q175

D214

E212

H228

D257

P258

D259

R251

T226

Q217

Figure S2. Aligned optimized (a) **Res16-QA-R1** (cyan), (b) **Res20-QA-R1** (yellow) and **Res20-QB-R1** (cyan), (c) **Res21-QA-R1** (yellow) and **Res21-QB-R1** (cyan), and (d) **Res23-QA-R1** (cyan) to each of the model of X-ray crystal structure (magenta). Waters are shown in spheres. **Res21-EA-R1** is shown (in d, green) with waters are shown in green spheres green.
